# Fairness in the prediction of acute postoperative pain using machine learning models

**DOI:** 10.3389/fdgth.2022.970281

**Published:** 2023-01-11

**Authors:** Anis Davoudi, Ruba Sajdeya, Ron Ison, Jennifer Hagen, Parisa Rashidi, Catherine C. Price, Patrick J. Tighe

**Affiliations:** ^1^ Department of Anesthesiology, University of Florida College of Medicine, Gainesville, FL, United Sates; ^2^ Department of Epidemiology, University of Florida College of Public Health and Health Professions, Gainesville, FL, United States; ^3^ Department of Orthopedic Surgery, University of Florida College of Medicine, Gainesville, FL, United States; ^4^ Department of Biomedical Engineering, University of Florida Herbert Wertheim College of Engineering, Gainesville, FL, United States; ^5^ Department of Clinical and Health Psychology, University of Florida College of Public Health and Health Professions, Gainesville, FL, United States

**Keywords:** algorithmic bias, machine learing, clinical decision support systems, postoperative pain, orthopedic procedures

## Abstract

**Introduction:**

Overall performance of machine learning-based prediction models is promising; however, their generalizability and fairness must be vigorously investigated to ensure they perform sufficiently well for all patients.

**Objective:**

This study aimed to evaluate prediction bias in machine learning models used for predicting acute postoperative pain.

**Method:**

We conducted a retrospective review of electronic health records for patients undergoing orthopedic surgery from June 1, 2011, to June 30, 2019, at the University of Florida Health system/Shands Hospital. CatBoost machine learning models were trained for predicting the binary outcome of low (≤4) and high pain (>4). Model biases were assessed against seven protected attributes of age, sex, race, area deprivation index (ADI), speaking language, health literacy, and insurance type. Reweighing of protected attributes was investigated for reducing model bias compared with base models. Fairness metrics of equal opportunity, predictive parity, predictive equality, statistical parity, and overall accuracy equality were examined.

**Results:**

The final dataset included 14,263 patients [age: 60.72 (16.03) years, 53.87% female, 39.13% low acute postoperative pain]. The machine learning model (area under the curve, 0.71) was biased in terms of age, race, ADI, and insurance type, but not in terms of sex, language, and health literacy. Despite promising overall performance in predicting acute postoperative pain, machine learning-based prediction models may be biased with respect to protected attributes.

**Conclusion:**

These findings show the need to evaluate fairness in machine learning models involved in perioperative pain before they are implemented as clinical decision support tools.

## Introduction

Acute postoperative pain is a significant public health problem. Eighty percent of surgical patients report experiencing postoperative pain, with as high as 88% of those reporting moderate or higher levels of pain (1, 2). Severe acute postoperative pain is associated with the development of persistent postoperative pain, although the nature of this relationship remains unclear (3–5). Poorly managed acute postoperative pain may lead to adverse outcomes, including lower patient satisfaction with pain management, delayed inpatient recovery and discharge, increased costs of care, chronic pain, inappropriate opioid prescribing, increased risk of opioid misuse and opioid use disorder, overdose, and death (2, 6–10).

One reason for suboptimal pain management is the difficulty in predicting severe acute postoperative pain. A preoperative prediction model for acute postoperative pain could, for instance, suggest a preoperative application of regional anesthetic techniques in patients whose surgical procedures may not otherwise qualify for such therapies based on local procedure-based heuristics. Over the past several decades, numerous models have been proposed to understand patient and procedural risk factors for severe acute postoperative pain (11–13). Although these models helped determine relevant predisposing and precipitating factors of moderate to severe postoperative acute pain, they often incorporated features that required extra clinical assessments, such as pain catastrophizing, anxiety, and functional disability tests (11, 14–16). Additionally, most previous research in this domain has focused on determining risk factors for postoperative pain using statistical methodology. Prior work suggests that given similar features, machine learning models can outperform linear statistical models in classifying outcomes related to postoperative pain (17, 18). Previous work using machine learning to predict pain with perioperative data shows promising performance with an area under the curve (AUC) of 0.70 for predicting acute postoperative pain (19).

Although machine learning methods have significantly improved the accuracy of predictions, questions remain concerning their interpretability and fairness. Those aspects are especially important for future implementation and translational research. Previous research using machine learning in healthcare primarily focused on the model's overall performance, which was evaluated based only on how well the model predicted the decided outcome for the test dataset. Recently, there has been growing concern about the performance of these models among underrepresented and marginalized groups that may not be well represented in the dataset used for training the model. This is a crucial consideration for physicians applying population-level models on individual patients from underrepresented backgrounds who may ask how well such models apply to them personally. Here, concerns over machine learning “fairness” refer to the algorithmic bias in machine learning approaches, where the developed models will systematically predict an outcome more likely for one group than another, especially when these groupings are based on sensitive attributes that should not be correlated with the outcomes (e.g., ethnicity, gender, disability status). Using a model with a strong performance in the general population but that is biased against unprivileged groups might be harmful to patients in the unprivileged subcohorts.

To date, there have been no formal assessments of fairness in machine learning models used to predict postoperative pain. This retrospective cohort analysis examines the fairness of a best-in-class machine learning model that predicts acute postoperative pain among patients presenting for orthopedic surgery. We hypothesized that even in models that performed well overall in classification accuracy and the AUC, select population subgroups may suffer from much poorer performance regarding acute postoperative pain risk stratification.

## Materials and methods

The study protocol was approved by the University of Florida Institutional Review Board (IRB #201601338), which waived informed consent. This retrospective single-center machine learning study was designed and conducted according to Guidelines for Developing and Reporting Machine Learning Predictive Models in Biomedical Research: A Multidisciplinary View (20).

### Dataset

The retrospective cohort consisted of adult surgical patients undergoing orthopedic surgery at the University of Florida Health system/Shands Hospital between June 1, 2011, and June 30, 2019, and residing in Florida at the time of surgery. Orthopedic surgical procedures are among the surgical procedures with the highest postoperative pain (21–23). The cohort of patients undergoing orthopedic surgery was not constrained to any specific sociodemographic group. Data were provided by the UF Health Integrated Data Repository *via* an honest data broker; all variables were validated *via* a continuous quality control process.

Our primary diagnostic outcome was mean pain on postoperative day 1 (POD1; during the day after the surgery). We used clinical pain intensities assessed using the Defense and Veterans Pain Rating Scale (DVPRS, ranging from 0 to 10) (24) and entered into the electronic health record (EHR) system as part of routine clinical care. Notably, the EHR implementation contains user prompts providing instruction in the bedside application of the DVPRS. The mean of all numerical pain scores of the patients on POD1 was calculated and dichotomized into a binary outcome: no pain or mild pain class (discussed as “low pain” in subsequent sections; pain scores 0–4) and moderate or severe pain class (discussed as “high pain”; pain scores >4). The observation unit for the outcome and predictors was patient-based. This threshold was based on prior work establishing cutpoints for mild pain intensity (25–28).

Pain management guidelines at this institution have been developed in concert with the surgical service and acute pain medicine service. These guidelines heavily emphasize multimodal analgesic strategies with regular use of preoperatively placed continuous catheter-based peripheral, paravertebral, and neuraxial regional anesthesia by faculty with fellowship training in regional anesthesia and acute pain medicine. All patients who receive blocks are reviewed upon arrival to the recovery room or intensive care unit, and block adjustments or additions are made accordingly. The central tenants of these guidelines have largely remained intact over the past decade, with annual review and updates as needed. Additional details of this process have been published previously (16).

We included common sociodemographic and clinical variables routinely available for surgical patients, including age, sex, race, ethnicity, marital status, body mass index (BMI), language, health literacy, insurance, area deprivation index (ADI) (29) of patient's residence, diagnosis categories, current procedure terminology (CPT) category, combined comorbidity score (30, 31), and the American Society of Anesthesiologists physical status (ASA-PS) classification (32). Health literacy was determined using the Rapid Estimate of Adult Literacy in Medicine-Revised (REALM-R) assessment (33), and patients who could correctly pronounce all of the eight proposed words were recorded as having adequate health literacy. Procedure and diagnosis categories were determined *via* the clinical classification software (CCS), which is a Healthcare Cost and Utilization Project (HCUP) research tool, using patients' CPT codes and International Classification of Diseases (ICD 9 and 10) codes (34–36). These variables were chosen given their general widespread availability in administrative datasets. CPT categories are referred to as CCS-CPT in this article.

We used “sociome” and “sf” packages in R for extracting patient ADI information (37, 38). ADI scores were extracted from the “sociome” R package for each census tract in the state of Florida using 15 variables from the American Community Survey for the year 2019 (5-year data) (Supplementary Table S1). The ADI encompasses education, employment, poverty, and environment indicators in the census tract, with higher values showing worse neighborhood deprivation. We used the shapefiles of census tract borders available from the US Census Bureau (39). Latitude and longitude coordinates of the patients' residences at the time of surgery were spatially joined with the polygons of the census tract borders. The extracted neighborhoods' US Census Bureau geographic identifiers (GEOIDs) were used to find neighborhood tract attributes from the census data tables. ADI is a continuous variable and was used as such in the prediction models. We transformed its value into tertiles for our analysis of fairness.

### Machine learning models and statistical considerations

**Figure 1:**
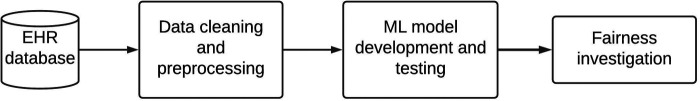
Analytical workflow. EHR, electronic health records; ML, machine learning.

Figure 1 shows the analytical workflow. The variables used in POD1 pain prediction models were summarized and compared between the two patient groups using the student t-test for continuous variables and chi-squared test for categorical variables.

We used the CatBoost machine learning classification models to predict pain using EHR data. We used fourfold nested cross-validation for parameter tuning using AUC as the loss function and reported models' performance as the mean (SD) of the model performance metrics on the unseen test data for each of the four outer folds (internal validation with threefold cross-validation with patient-based split). We reported the model performance in terms of accuracy and balanced accuracy, AUC, precision, recall, and F1-score. We used the “CatBoost” library for developing CatBoost models. We did not use stratification based on the outcome in training the models because the outcome was not significantly imbalanced. Similarly, we did not use stratification based on any categorical variables in training the model because the aim of the study was to investigate the severity of algorithmic bias in the machine learning models developed for pain prediction used as decision support tools, which usually relay data reflective of the real patients' data. We reported the ranking of the variable importance in the model's training using variable importance extracted from the model, based on the change in the loss function. Observing the variable importance ranking is helpful for feature selection and informed dimensionality reduction. Information on variable importance ranking in the model also provides insight into the data and the model. A higher importance ranking of protected attributes may cause concern for fairness in that attribute. Further details of data cleaning and preprocessing steps and model developments are reported in the Supplementary Methods, Supplementary Tables S2, S3 and Figure S1.

### Fairness

#### Investigating bias

We studied model bias for the following sociodemographic attributes: age, sex, race, language, health literacy, ADI, and insurance type. In this context, the privileged group was defined as subcohorts with a lower risk of adverse clinical outcomes. The unprivileged groups were determined as subcohorts with a higher risk of adverse clinical outcomes. Table 1 shows the classes of protected attributes and their corresponding privileged and unprivileged values. We used the “Dalex” library in Python to evaluate model fairness (40). In investigating the fairness of the classifier concerning each of the attributes mentioned, several model performance metrics were calculated and compared between the privileged and unprivileged subcohorts. For each protected attribute, model performance was calculated based on the performance metrics defined in Table 2 for each unprivileged subcohort separately and compared to the model's performance for the privileged group (41, 42).

**Table 1:** Privileged and unprivileged values of the protected attributes studied.

Protected attribute	Privileged value	Unprivileged value (s)
Age	Younger adults (40 years old or younger)	Middle-aged (between 40 and 65 years old)
		Older adults (65 years or older)
Sex	Male	Female
Race	White	Non-White
Language	English speaking	Non-English speaking
Health literacy	Adequate	Limited
ADI	Lowest ADI tertile	Middle ADI tertile
		Highest ADI tertile
Insurance type	Private (“Blue Cross”, “Commercial”, “Managed Care”, “Medicare HMO”, “Workers Com”, and “Federal” insurances)	Public (“Medicaid”, “Medicaid HMO”, “other”, and “self-pay” insurances)
		Medicare (Medicare insurance)

ADI, area deprivation index.

**Table 2:** Model performance metrics definitions.

Performance metric	Formula	Machine learning concept	Fairness metric
True positive rate (TPR)^a^	$\frac{\mathrm{TP}}{\mathrm{TP}+\mathrm{FN}}$	the probability that an actual positive will test positive	Equal opportunity
False positive rate (FPR)^b^	$\frac{\mathrm{FP}}{\mathrm{FP}+\mathrm{TN}}$	the probability that an actual negative will test positive	Predictive equality
Positive predictive value (PPV)	$\frac{\mathrm{TP}}{\mathrm{TP}+\mathrm{FP}}$	the probability that a positive test is actually positive	Predictive parity
Accuracy	$\frac{\mathrm{TP}+\mathrm{TN}}{\mathrm{TP}+\mathrm{FP}+\mathrm{TN}+\mathrm{FN}}$	number of true predictions/numbers of prediction	Overall accuracy equality
Statistical parity (STP)	$\frac{\mathrm{TP}+\mathrm{FP}}{\mathrm{TP}+\mathrm{FP}+\mathrm{TN}+\mathrm{FN}}$	the probability of positive prediction for either class	Statistical parity

TP, true positive rate; FN, false negative; TN, true negative; FP, false positive.

^a^ Also known as sensitivity and recall.

^b^ FPR: 1-True negative rate.

More specifically, we calculated the ratio (Equation 1) for each unprivileged class and each model performance metric. The closer this ratio is to 1, the fairer the model performance. With *ɛ* a value between 0 and 1, we used the value of 0.8 as a threshold to determine bias in our models’ performance as a threshold for bias in other domains (known as the “80% rule”) (43). *ɛ* value of 0.8 resulted in an acceptable range of model performance ratio between 0.8 and 1.25, meaning that if the ratio defined in Equation 1 was between 0.8 and 1.25, the model was reported to not be biased for that metric and that attribute class:

(1) $$\forall_{i\in \left\{a,\;b,\;\ldots ,\;z\right\}}\;\;\;\;\;\;\;\;\varepsilon <\frac{\mathrm{metri}c_{i}}{\mathrm{metri}c_{\;\mathrm{privileged}}}<\frac{1}{\varepsilon }$$

#### Mitigating bias

To address the algorithmic bias in the prediction models, we used a reweighing approach to adjust the weight of observations in each attribute-outcome combination in training the model and compared the bias in the new models to the base models. In this approach, a new model was built using the observation weights defined based on the number of observations (patients) in each unprivileged and privileged group.

Data preparation and analysis were performed in R V4.0.0 and Python V3.8.5.

## Results

### Dataset

**Figure 2:**
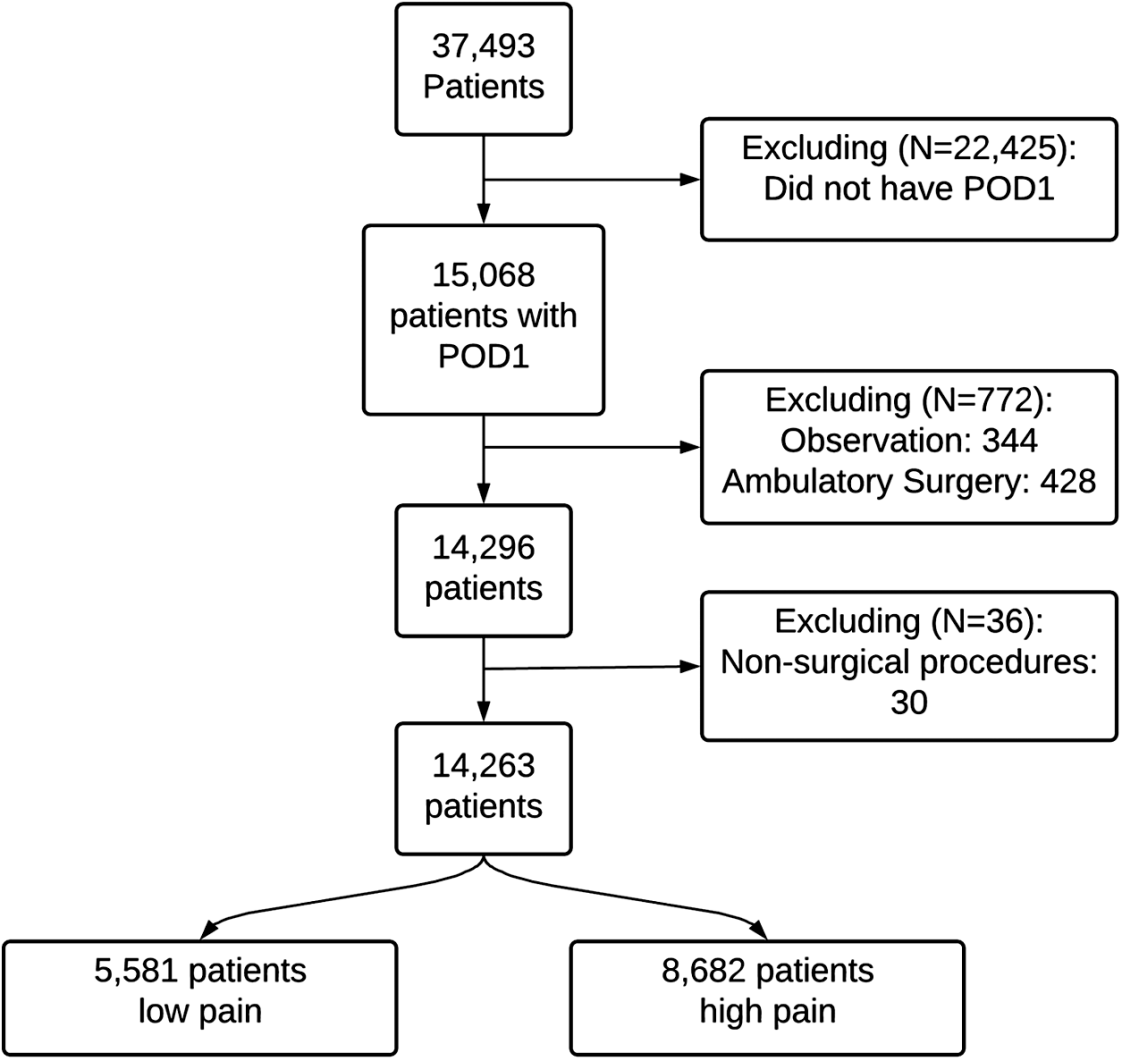
Cohort selection process. POD1, postoperative day 1.

**Figure 3:**
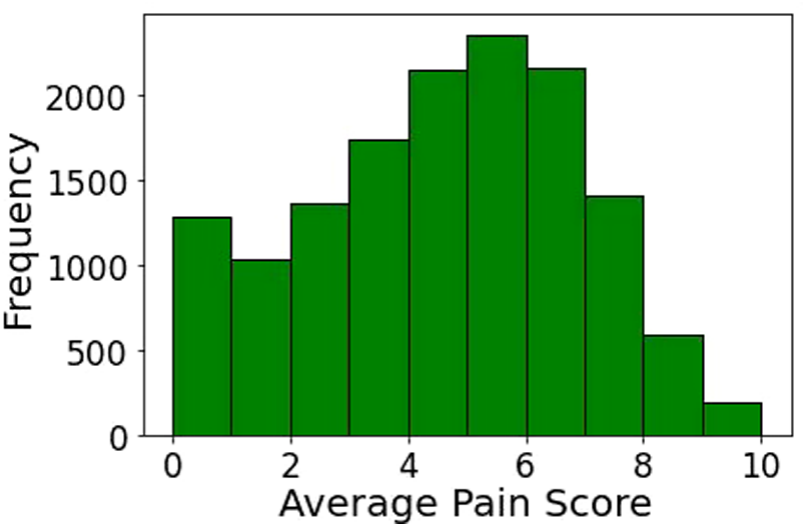
Distribution of mean pain score on postoperative day 1.

Between June 1, 2011, and June 30, 2019, 37,493 patients had orthopedic surgery at the University of Florida Health Hospital. Figure 2 shows the cohort selection process in the study. Our final cohort included 14,263 patients. The mean (SD) age was 60.72 (16.03) years and 53.87% were women (Supplementary Table S4). Figure 3 shows the distribution of mean pain scores on POD1. There were 5,581 (39.13%) patients with no pain to low mean pain (low pain) and 8,682 (60.87%) patients with moderate to severe pain (high pain) on POD1. Sex, ethnicity, health literacy, and the combined comorbidity score were not significantly different between the two groups. Patients with low pain on POD1 were generally older, predominantly White, and married, and they generally had lower BMIs, better socioeconomic status in terms of ADI, and higher ASA-PS class. The distribution of only four diagnosis categories, (1) coma, stupor, and brain damage; (2) other gastrointestinal disorders; (3) anxiety disorders; and (4) mood disorders), and CCS-CPT were also significantly different between the two patient groups. We kept diagnoses that were present in at least 1% of the patients (101 diagnoses).

### Machine learning models

CatBoost classifiers used were able to predict mean pain on POD1 for all patients with accuracy 0.67 and AUC 0.71 (Supplementary Table S5, calibration plot reported in Supplementary Figure S2). Supplementary Figure 3 shows the ranking of the importance of the top 20 variables for the CatBoost model using the importance calculated in the model. Age (mean importance: 50.15) and insurance (mean importance: 14.51) were the most important variables. The distribution of age and insurance were also significantly different between the patient group with high POD1 pain and the patient group with low POD1 pain (Table 3). The rankings of ASA-PS, ADI, CCS-CPT category, and marital status were similar. Sex, BMI, health literacy, ethnicity, race, language, combined comorbidity score, and diagnosis categories had less important contributions to the model.

**Table 3:** Distribution of outcome in each protected attribute level.

Protected attribute levels	Low pain (*N* = 5,581)	High pain (*N* = 8,862)	*P*-value
Age			**<0** **.** **0001**
Younger adult	350 (18.65%)	1,527 (81.35%)	
Middle-aged	1,673 (28.98%)	4,100 (71.02%)	
Older adult	3,558 (53.80%)	3,055 (46.20%)	
Race			**<0** **.** **0001**
White	4,610 (40.42%)	6,795 (59.58%)	
Non-White	786 (32.29%)	1,648 (67.71%)	
Sex			0.6105
Male	2,590 (39.36%)	3,990 (60.64%)	
Female	2,991 (38.93%)	4,692 (61.07%)	
Health literacy			0.4179
Adequate	3,957 (38.71%)	6,266 (61.29%)	
Limited	494 (37.51%)	823 (62.49%)	
ADI			**<0** **.** **0001**
Lowest tertile	2,200 (46.61%)	2,520 (53.39%)	
Middle tertile	1,895 (38.56%)	3,020 (61.44%)	
Highest tertile	1,485 (32.10%)	3,141 (67.90%)	
Language			**0** **.** **0099**
English speaking	5,472 (38.99%)	8,562 (61.01%)	
Non-English speaking	109 (47.60%)	120 (52.40%)	
Insurance type			**<0** **.** **0001**
Private	2,394 (39.20%)	3,713 (60.80%)	
Public	463 (17.99%)	2,110 (82.01%)	
Medicare	2,724 (48.79%)	2,859 (51.21%)	

ADI, area deprivation index.

Bold values indicate *p*-values < 0.05.

### Fairness

#### Investigating bias

Model biases were examined for the classifier with the best performance. We evaluated the fairness of the models for the following attributes: age, sex, race, speaking language, ADI tertile, health literacy, and insurance type.

Table 3 shows the distribution of the outcome in each level of the protected attributes. Among the patients in the younger adult age group, more than 80% had high pain on POD1, whereas 71.02% had high pain in the middle-aged age group and 46.20% had high pain in the older adult age group. The percentage of high pain patient groups increased with higher ADI tertile, and it was higher among English-speaking patients. Although the Medicare insurance group had a similar percentage of high pain and low pain prevalence, more than 80% of public insurance patients were in the high pain groups. The percentage of high and low pain prevalence was not significantly different between the different classes of sex and health literacy attributes.

Table 4 shows the models' performance in different subcohorts considering single protected attributes in each analysis. When considering age alone, the prediction model was not fair; bias was detected in all five examined metrics. Performance was unbiased for the middle-aged group and biased for the older adult group compared to the model performance for the younger adult group (the privileged group). We considered “younger adults” the privileged group because younger patients generally have a lower risk of adverse clinical outcomes. However, in our dataset, the younger adult group was in the minority (13.16% of the dataset). Although the desired performance metrics [true positive rate (TPR), accuracy (ACC), and positive predictive value (PPV)] were highest for this group, the undesired performance metric [false positive rate (FPR)] was also the highest for this group (FPR = 1.00). This means that most negative events (low pain) of the younger adult patients were predicted to be the positive event (high pain). Although the older adult age group was in the majority in our dataset (46.36% of the whole dataset), the models' performance in terms of TPR, ACC, and PPV was significantly worse (outside of the 0.8–1.25 range) for the older adult age group than the younger adult age group. However, their FPR and statistical parity (STP) were also lower. Lower FPR meant that there were fewer true negative samples misclassified as positive (high pain).

**Table 4:** Model performance for each unprivileged group compared to privileged group.

Attribute	Group	Equal opportunity ratio	Accuracy equality ratio	Predictive parity ratio	Predictive equality ratio	Statistical parity ratio
Age	Younger adult^a^ (13.16%)	1.00	0.81	0.81	1.00	1.00
	Middle-aged (40.48%)	0.98	0.88	0.89	0.92	0.96
	Older adult (46.36%)	**0** **.** **52**	**0** **.** **76**	**0.74**	**0** **.** **29**	**0** **.** **40**
Race	White^a^ (79.96%)	0.80	0.68	0.71	0.50	0.68
	Other (17.07%)	1.13	1.05	1.05	**1** **.** **36**	1.22
Sex	Male^a^ (46.13%)	0.83	0.70	0.72	0.51	0.70
	Female (53.87%)	0.98	0.98	0.99	1.05	1.01
Health Literacy	Adequate^a^ (71.67%)	0.83	0.68	0.71	0.55	0.72
	Limited (9.23%)	0.98	1.03	1.05	0.87	0.95
ADI	Lowest tertile^a^ (33.33%	0.69	0.65	0.67	0.39	0.55
	Middle tertile (33.33%)	**1.23**	1.07	1.07	**1** **.** **37**	**1** **.** **32**
	Highest tertile (33.33%)	1.32	1.10	1.10	**1** **.** **76**	**1** **.** **53**
Language	English^a^ (98.39%)	0.82	0.69	0.71	0.53	0.71
	Non-English (1.61%)	0.91	0.96	0.92	0.84	0.85
Insurance	Private^a^ (42.82%)	0.87	0.66	0.67	0.66	0.79
	Public (18.04%)	1.14	1.24	1.23	**1** **.** **45**	**1** **.** **26**
	Medicare (39.14%)	**0** **.** **73**	0.98	1.00	**0** **.** **50**	**0** **.** **61**

ADI, area deprivation index.

The performances with bias detected are shown in bold. Younger adult is defined as younger than 40 years; middle-aged is between 40 and 64 years; and older adult is older than 64 years. The numbers in parentheses show the percentage of each subcohort in the dataset.

^a^ The privileged group for each attribute.

The models were biased regarding race only in the FPR metric (non-White patients had 1.39 times higher FPR than White patients). The models had bias with respect to ADI in three of the five examined metrics: TPR, FPR, and STP. The patients residing in the neighborhoods with ADI in the highest tertile had 1.38 times higher TPR than those residing in the lowest-tertile ADI. Patients living in neighborhoods with middle- and highest-tertile ADI also had higher FPR and STP than those residing in neighborhoods with the lowest-tertile ADI (Table 4). Higher ADI tertile groups had a larger percentage of patients in the high pain group than patients in the low pain group patients (Table 3). In three out of the five examined metrics, the model performance was also biased regarding patient insurance type. We did not find any bias in the models with respect to sex, health literacy, and language.

We also examined fairness for attribute pairs (Supplementary Table S6), but we did not investigate a larger number of combinations (more than two attributes together) because when considering more attributes together, the number of combinations would increase significantly. The size of minority groups would also become very small for most of the groups.

For attribute pairs where no bias was detected for either of the attributes (language-health literacy, sex-health literacy, sex-language), there was almost no bias in their composite subcohorts (except for in TPR and STP for the female-non-English–speaking subcohort, compared to the male-English–speaking subcohort).

In attribute pairs where the models were not biased in terms of one of the attributes, the same bias patterns as in the attribute with the bias generally persisted. For example, for the age-health literacy attribute pairs, the model performance was biased regarding the older adult patient group with both adequate and limited health literacy in a similar direction as the model bias regarding the older adult group alone.

In attribute pairs where the models were biased in both attributes, the bias patterns did not necessarily follow the same patterns as every single attribute if the bias direction (larger or smaller than the privileged group) differed in the two attributes. For example, predictive equality and STP ratios were larger than 1.25 for middle tertile ADI; equal opportunity, predictive equality, and STP ratios were larger than 1.25 for the highest tertile ADI than the lowest tertile ADI. Predictive equality and STP ratios were larger than 1.25 for the public insurance type but smaller than 0.8 for Medicare when compared to the private insurance type. When observing the model bias for insurance type and ADI tertile attribute pairs, predictive equality and STP ratios were larger than 1.25 for private insurance type and highest tertile groups and public insurance type and all ADI tertiles. However, this direction was reversed for the Medicare insurance type subcohort, with some bias detected in Medicare insurance type and lowest and middle ADI tertile.

### Mitigating bias

To reduce the algorithmic bias in our models, we examined the effect of reweighing the observations (patients) with respect to their belonging to each subcohort created based on protected attribute classes. Supplementary Figures S4–S11 show the effect of reweighing on bias in the models' performance. The reweighing did not change this case for sex and health literacy attributes, which did not have any bias in the model performance. However, for the language attribute, reweighing the observations based on the language group caused bias in a case where there was no bias before. (The fairness metrics for the non-English–speaking subcohort deteriorated for the equal opportunity ratio from 0.91 to 1.35, for the overall accuracy ratio from 0.96 to 1.30, and for the predictive equality from 0.84 to 0.29.). Although reweighing might help reduce the bias, as is the case for the ADI attribute (Supplementary Figure S8), reweighing based on one attribute can hurt the model fairness in terms of other protected attributes. For example, prediction models reweighed based on the ADI tertile label added to the algorithm bias in the models’ performance with respect to race (Supplementary Figure S11). Supplementary Figure S6 shows that reweighing the observations using the race attribute improved model fairness regarding race. In contrast, Supplementary Figure S11 shows that reweighing based on the ADI attribute exacerbated the algorithm bias regarding race. In comparing the two figures, we can see that not only is the bias in predictive equality not resolved, but the model has also become biased in terms of STP.

## Discussion

We used machine learning models to predict acute postoperative pain in a retrospective study of a single-center cohort of orthopedic surgical patients. Patients' age and insurance type were the two most important variables for training the CatBoost model. We also examined the prediction models with respect to bias regarding multiple attributes, including age, sex, race, health literacy, ADI, language, and insurance type. The models did not show any significant bias regarding sex, language, and health literacy, although the unprivileged groups for both language and health literacy were in a clear minority. Bias was found for variables where the distributions of the outcome labels between the privileged and unprivileged subcohort were significantly different, except for the speaking language protected attribute, which had a very low variable importance ranking (Table 3 and Supplementary Figure 3).

The model was biased against the patients with other (non-White) races in terms of FPR. FPR was 1.36 times higher for races other than White, meaning that they would incorrectly be labeled as the positive event (high pain in our study) 1.36 times more often.

The bias detected with respect to age showed that even though the privileged class (younger adult) was in the minority in our dataset (13.16% of the study cohort), it had higher TPR, ACC, and PPV than the middle-aged and older adult groups. Put another way, even though the model was trained and evaluated with a larger number of older adult patients than younger adult patients, the model performance was more unsuccessful among older adults in predicting high pain (positive event). This is somewhat counterintuitive given that an oft-cited reason for model unfairness for a minority subgroup is their underrepresentation in the training data (44–46). One reason for these results could be that the high pain group was significantly younger (on average approximately 10 years) than the low pain. This difference might have made the model more inclined to predict high pain labels for the younger adult age group, particularly as age was the most important variable for the model training. This rationale would comport with prior literature associating younger age with greater acute postoperative pain intensity (12, 22, 47, 48). This is further supported by the high FPR of 1.00 for the younger adult group, which along with the TPR of 1 showed that all younger adult patients were predicted to have high pain, whereas 18.65% of them were in the low pain group. In the older adult group, the ratio of high and low pain labels was almost the same (46.20% in the high pain group), probably leading to lower model performance in this group because the age variable was not very informative anymore. Similarly, insurance was the second most important variable in the developed pain prediction models, and its distribution was significantly different between the two pain groups (Supplementary Table S4). Moreover, Medicare patient groups had almost the same chance of experiencing high or low pain during their first postoperative day, whereas 60.80% of patients using private insurance and 82.01% of patients using public insurance were in the high pain group (Table 3).

The model had a higher correct prediction of the positive class (high pain) for the unprivileged groups for the ADI attribute than the privileged (lowest-tertile) ADI group. The rates of the positive event (high pain) in the two unprivileged subcohorts were higher (61.44% and 67.90%, respectively) than the 53.39% in the lowest-tertile ADI group. However, these two groups also had higher FPR and STP than the lowest-tertile ADI.

The bias detected in attribute-pair analyses followed patterns that were similar to the bias of the specific attributes in the pair. For example, almost all attribute pairs, including age, were biased regarding the older adult group, similar to the bias pattern detected in the single attribute analysis. When the model was biased regarding both attributes in the pair, the bias pattern would generally be more substantial. However, if the biases in a metric for two protected attributes were in opposite directions, they might cancel each other to some extent. For example, the higher increase in the highest ADI tertile seems to have canceled out the decreased direction of Medicare insurance class. There were few exceptions to these two patterns, such as the non-English–speaking and middle-aged group and non-English–speaking and female group. One potential reason for these exceptions might be the much smaller size of the subcohorts (<1% of the dataset). Sometimes, the much smaller size of the subcohort seemed to lead to bias as well. For example, although the model was not biased in terms of sex or language attributes, the model was biased against the subcohort of female and non-English–speaking patients (0.86% of the whole cohort) with lower TPR, FPR, and STP. However, the model was not biased against the subcohort of male and non-English–speaking patients (0.74% of the entire cohort), which could partially be because one of the attributes was from the privileged group (male).

Reweighing the prediction models based on each protected attribute helped reduce the bias for some cases (e.g., ADI), but it introduced bias in some other cases where there was no bias (language). Speaking language was not an important variable in training the model, and this change in the model bias might have resulted from any potential change in the distribution of other attributes in the training data. Similarly, reweighing based on one attribute can hurt the model fairness in terms of other protected attributes, which might have resulted from potential changes in the distribution of other attributes in the training data.

We also did not investigate the effect of reweighing for addressing bias in attribute pairs because the combination of two protected attributes and the outcome class would have created too many subcohorts for analysis.

This is the first study to assess the fairness performance of machine learning-based pain prediction models in different subcohorts, considering several protected attributes, including age, sex, race, insurance type, socioeconomic status at neighborhood level (ADI), language, and health literacy. The bias we detected in the developed models clearly shows that despite the promising overall performance of the model (AUC, 0.71; balanced accuracy, 0.64), the performance suffers significantly for some of the subcohorts.

One implication of these findings is that machine learning-based pain prediction models need to be validated in different subcohorts before they are used in practice. Another possible direction is to develop separate pain prediction models for each subcohort (hierarchical stratification). Our findings showed that unprivileged subcohorts experienced more bias in pain prediction based on age, ADI, and insurance types attributes. These findings show the need to assess and address the algorithmic bias in the prediction systems developed as decision support systems in the healthcare outcomes domain. Implementing fair systems to predict postoperative pain helps ensure the patients, surgical team, and healthcare team have a more accurate picture of a patient's risk of high postoperative pain.

Machine learning approaches have increasingly been used to produce robust decision support systems in healthcare research and clinical applications. Although fairness in healthcare services for different demographic populations has been discussed previously (49–52), the issue of fairness in the healthcare applications of machine learning has come into focus only recently (45, 53–58). Recent attention to fairness in machine learning, particularly machine learning approaches in healthcare, has been placed on the performance of the developed models in subcohorts that are differentiated based on protected attributes. To date, most of the research on bias in machine learning in healthcare has been focused on bias against non-White races. For example, Park et al. (54) showed that machine learning-based approaches using the IBM MarketScan Medicaid Database to predict postpartum depression and mental health service use were biased against Black women. The study also reported that reweighing race (the protected attribute) improved the model's fairness in terms of disparate impact (similar to STP) and equal opportunity difference without compromising the model performance in terms of balanced accuracy. Bias toward non-White races has been shown in other healthcare algorithms as well (53).

This was a retrospective study, and consequently, we could not include many of the factors relevant to the prediction of acute postoperative pain, such as preoperative pain, anxiety, and pain catastrophizing (11). However, developing acute postoperative pain prediction models using real-world data may be more helpful in translating such models to pragmatic clinical decision support systems.

Our investigation used pain intensity as the primary outcome. Pain intensity is a common outcome used for assessing postoperative pain experience in the perioperative pain management literature, and it is amenable to classification exercises given the nature of its measurement (59). However, it is important to note several related aspects of the perioperative pain experience including analgesia, function (e.g., mobility, return of bowel function), pain quality, and the potential for partial causative relationships with postoperative complications (60). Future work is necessary to classify pain-related outcomes across a multiobjective front.

Another limitation in supervised machine learning research stems from feature definition and capturing. Our preprocessing steps for missing values might affect the prediction of patients with missing values for some predictors. This effect might vary based on whether the missingness was informative or random. In our cross-validation, to prevent any data leakage from testing data to the model development, we used the information extracted from training data when imputing missing variables in the test data, as described in Supplementary Content. Regrouping levels with a smaller size for factors such as marital status is another limitation of the feature preprocessing. Limited degrees of granularity for variables such as sex (female or male) and health literacy (limited testing) affect the usefulness of captured information. Another limitation in the study was that some of the categorical variables had small classes for some levels. Because we treated the pain prediction model as a decision support tool, we did not perform any further preprocessing on such categorical variables. Another related limitation of the study was that some of the subcohorts in the fairness analyses were very small (less than 1% of the whole cohort). The small size of some subcohorts might reduce the robustness of the results; however, this issue is inherent to real-world clinical studies. One primary reason for bias in machine learning is the insufficient representation of unprivileged groups in the training dataset used to develop the model. Moreover, our dataset was obtained from a single-center cohort and is reflective of the population of orthopedic surgery patients in the state of Florida, limiting the generalizability of the findings.

We also used a threshold of 0.8 for determining bias when comparing the model's performance for the unprivileged subcohorts to its performance for the privileged subcohort. This threshold was adopted from the hiring practices, and a more appropriate bias threshold for the differences in machine learning model performance in the healthcare domain, and particularly pain prediction for different subcohorts, needs to be investigated and established.

The detected bias in our prediction models (whose overall performance is similar to the recent models in the published literature) shows the need to examine the diversity in different attributes of the training dataset and the model performance in unprivileged subcohorts before implementing and using decision support systems to predict acute postoperative pain.

## Data Availability

The original contributions presented in the study are included in the article/**Supplementary Material**, further inquiries can be directed to the corresponding author/s.

## References

[1] Simon LS. Relieving pain in America: a blueprint for transforming prevention, care, education, and research. J Pain Palliat Care Pharmacother. (2012) 26:197–8. 10.3109/15360288.2012.678473

[2] Buvanendran A Fiala J Patel KA Golden AD Moric M Kroin JS. The incidence and severity of postoperative pain following inpatient surgery. Pain Med. (2015) 16:2277–83. 10.1111/pme.12751 25917518

[3] Kehlet H Jensen TS Woolf CJ. Persistent postsurgical pain: risk factors and prevention. Lancet. (2006) 367:1618–25. 10.1016/S0140-6736(06)68700-X 16698416

[4] Buvanendran A Della Valle CJ Kroin JS Shah M Moric M Tuman KJ Acute postoperative pain is an independent predictor of chronic postsurgical pain following total knee arthroplasty at 6 months: a prospective cohort study. Reg Anesth Pain Med. (2019) 44:e100036. 10.1136/rapm-2018-100036 30770420

[5] Bruce J Quinlan J. Chronic post surgical pain. Rev Pain. (2011) 5:23–9. 10.1177/204946371100500306 26526062 PMC4590073

[6] Gan TJ Epstein RS Leone-Perkins ML Salimi T Iqbal SU Whang PG. Practice patterns and treatment challenges in acute postoperative pain management: a survey of practicing physicians. Pain Ther. (2018) 7:205–16. 10.1007/s40122-018-0106-9 30367388 PMC6251830

[7] Sinatra R. Causes and consequences of inadequate management of acute pain. Pain Med. (2010) 11:1859–71. 10.1111/j.1526-4637.2010.00983.x 21040438

[8] Neuman MD Bateman BT Wunsch H. Inappropriate opioid prescription after surgery. Lancet. (2019) 393:1547–57. 10.1016/S0140-6736(19)30428-3 30983590 PMC6556783

[9] Gan TJ. Poorly controlled postoperative pain: prevalence, consequences, and prevention. J Pain Res. (2017) 10:2287–98. 10.2147/JPR.S144066 29026331 PMC5626380

[10] Lovich-Sapola J Smith CE Brandt CP. Postoperative pain control. Surg Clin North Am. (2015) 95:301–18. 10.1016/j.suc.2014.10.002 25814108

[11] van Boekel RL Bronkhorst EM Vloet L Steegers MA Vissers KC. Identification of preoperative predictors for acute postsurgical pain and for pain at three months after surgery: a prospective observational study. Sci Rep. (2021) 11:1–10. 10.1038/s41598-021-95963-y 34385556 PMC8361098

[12] Schnabel A Yahiaoui-Doktor M Meissner W Zahn PK Pogatzki-Zahn EM. Predicting poor postoperative acute pain outcome in adults: an international, multicentre database analysis of risk factors in 50,005 patients. Pain Rep. (2020) 5:e831. 10.1097/pr9.0000000000000831 32766467 PMC7390596

[13] Coppes OJM Yong RJ Kaye AD Urman RD. Patient and surgery-related predictors of acute postoperative pain. Curr Pain Headache Rep. (2020) 24:12. 10.1007/s11916-020-0844-3 32072315

[14] Werner MU Mjöbo HN Nielsen PR Rudin Å Warner DS. Prediction of postoperative pain: a systematic review of predictive experimental pain studies. Anesthesiology. (2010) 112:1494–502. 10.1097/ALN.0b013e3181dcd5a0 20460988

[15] Kalkman JC Visser K Moen J Bonsel JG Grobbee ED Moons MKG. Preoperative prediction of severe postoperative pain. Pain. (2003) 105:415–23. 10.1016/s0304-3959(03)00252-5 14527702

[16] Vasilopoulos T Wardhan R Rashidi P Fillingim RB Wallace MR Crispen PL Patient and procedural determinants of postoperative pain trajectories. Anesthesiology. (2021) 134:421–34. 10.1097/aln.0000000000003681 33449996 PMC8726009

[17] Wu HY Gong CA Lin SP Chang KY Tsou MY Ting CK. Predicting postoperative vomiting among orthopedic patients receiving patient-controlled epidural analgesia using SVM and LR. Sci Rep. (2016) 6:27041. 10.1038/srep27041 27247165 PMC4887988

[18] Kumar V Roche C Overman S Simovitch R Flurin PH Wright T What is the accuracy of three different machine learning techniques to predict clinical outcomes after shoulder arthroplasty? Clin Orthop Relat Res. (2020) 478:2351–63. 10.1097/CORR.0000000000001263 32332242 PMC7491877

[19] Tighe PJ Harle CA Hurley RW Aytug H Boezaart AP Fillingim RB. Teaching a machine to feel postoperative pain: combining high-dimensional clinical data with machine learning algorithms to forecast acute postoperative pain. Pain Med. (2015) 16:1386–401. 10.1111/pme.12713 26031220 PMC4504764

[20] Luo W Phung D Tran T Gupta S Rana S Karmakar C. Guidelines for developing and reporting machine learning predictive models in biomedical research: a multidisciplinary view. J Med Internet Res. (2016) 18:e323. 10.2196/jmir.5870 27986644 PMC5238707

[21] Ekstein MP Weinbroum AA. Immediate postoperative pain in orthopedic patients is more intense and requires more analgesia than in post-laparotomy patients. Pain Med. (2011) 12:308–13. 10.1111/j.1526-4637.2010.01026.x 21143766

[22] Gerbershagen HJ Pogatzki-Zahn E Aduckathil S Peelen LM Kappen TH van Wijck AJ Procedure-specific risk factor analysis for the development of severe postoperative pain. Anesthesiology. (2014) 120:1237–45. 10.1097/aln.0000000000000108 24356102

[23] Gerbershagen HJ Aduckathil S van Wijck AJ Peelen LM Kalkman CJ Meissner W. Pain intensity on the first day after surgery: a prospective cohort study comparing 179 surgical procedures. Anesthesiology. (2013) 118:934–44. 10.1097/ALN.0b013e31828866b3 23392233

[24] Buckenmaier CC 3rd Galloway KT Polomano RC McDuffie M Kwon N Gallagher RM. Preliminary validation of the Defense and Veterans Pain Rating Scale (DVPRS) in a military population. Pain Med. (2013) 14:110–23. 10.1111/j.1526-4637.2012.01516.x 23137169

[25] Li KK Harris K Hadi S Chow E. What should be the optimal cut points for mild, moderate, and severe pain? J Palliat Med. (2007) 10:1338–46. 10.1089/jpm.2007.0087 18095813

[26] Serlin RC Mendoza TR Nakamura Y Edwards KR Cleeland CS. When is cancer pain mild, moderate or severe? Grading pain severity by its interference with function. Pain. (1995) 61:277–84. 10.1016/0304-3959(94)00178-H 7659438

[27] Alschuler KN Jensen MP Ehde DM. Defining mild, moderate, and severe pain in persons with multiple sclerosis. Pain Med. (2012) 13:1358–65. 10.1111/j.1526-4637.2012.01471.x 22925457 PMC3473137

[28] Gerbershagen HJ Rothaug J Kalkman C Meissner W. Determination of moderate-to-severe postoperative pain on the numeric rating scale: a cut-off point analysis applying four different methods. Br J Anaesth. (2011) 107:619–26. 10.1093/bja/aer195 21724620

[29] Singh GK. Area deprivation and widening inequalities in United States mortality, 1969–1998. Am J Public Health. (2003) 93:1137–43. 10.2105/AJPH.93.7.1137 12835199 PMC1447923

[30] Gagne JJ Glynn RJ Avorn J Levin R Schneeweiss S. A combined comorbidity score predicted mortality in elderly patients better than existing scores. J Clin Epidemiol. (2011) 64:749–59. 10.1016/j.jclinepi.2010.10.004 21208778 PMC3100405

[31] Sun JW Rogers JR Her Q Welch EC Panozzo CA Toh S Adaptation and validation of the combined comorbidity score for ICD-10-CM. Med Care. (2017) 55:1046–51. 10.1097/MLR.0000000000000824 29087983

[32] Doyle DJ Goyal A Bansal P Garmon EH. American society of anesthesiologists classification. In: Statpearls. Treasure Island, FL: StatPearls Publishing (2021). 28722969

[33] Bass PF 3rd Wilson JF Griffith CH. A shortened instrument for literacy screening. J Gen Intern Med. (2003) 18:1036–8. 10.1111/j.1525-1497.2003.10651.x 14687263 PMC1494969

[34] World Health Organization. International statistical slassification of siseases and health related problems. 10th Revision. Volume 1: Tabular List: World Health Organization (2004).

[35] Slee VN. International classification of diseases. Ninth revision. American College of Physicians. (1978). 10.7326/0003-4819-88-3-424 629506

[36] Agency for Healthcare Research and Quality. HCUP clinical classifications software (CCS) for services and procedures, v2020.1. Agency for Healthcare Research and Quality, Rockville, MD. (2020).

[37] Krieger N Dalton J Wang C Perzynski A. Sociome: Operationalizing social determinants of health data for researchers. (2021).

[38] Pebesma E. Simple features for R: standardized support for spatial vector data. R J. (2018) 10:439–46. 10.32614/RJ-2018-009

[39] Bureau USC. TIGER/Line Shapefiles. (2019).

[40] Baniecki H Kretowicz W Piatyszek P Wisniewski J Biecek P. Dalex: responsible machine learning with interactive explainability and fairness in Python. ArXiv. (2020).

[41] Verma S Rubin J. Fairness definitions explained. 2018 IEEE/ACM international workshop on software fairness (fairware): IEEE (2018).

[42] Wiśniewski J Biecek P. Fairmodels: a flexible tool for bias detection, visualization, and mitigation. arXiv. (2021):210400507.

[43] Feldman M Friedler SA Moeller J Scheidegger C Venkatasubramanian S. Certifying and removing disparate impact. Proceedings of the 21th ACM SIGKDD international conference on knowledge discovery and data mining (2015). p. 259–68. 10.1145/2783258.2783311

[44] Lee NT Resnick P Barton G. Algorithmic bias detection and mitigation: Best practices and policies to reduce consumer harms. Washington, DC, Unites States: Brookings Institute (2019).

[45] Panch T Mattie H Atun R. Artificial intelligence and algorithmic bias: implications for health systems. J Global Health. (2019) 9:010318. 10.7189/jogh.09.020318 PMC6875681 31788229

[46] Blattner L Nelson S. How costly is noise? Data and disparities in consumer credit. arXiv. (2021):210507554.

[47] Gagliese L Katz J. Age differences in postoperative pain are scale dependent: a comparison of measures of pain intensity and quality in younger and older surgical patients. Pain. (2003) 103:11–20. 10.1016/s0304-3959(02)00327-5 12749954

[48] Ip HY Abrishami A Peng Philip WH Wong J Chung F. Predictors of postoperative pain and analgesic consumption: a qualitative systematic review. Anesthesiology. (2009) 111:657–77. 10.1097/ALN.0b013e3181aae87a 19672167

[49] Marcelin JR Siraj DS Victor R Kotadia S Maldonado YA. The impact of unconscious bias in healthcare: how to recognize and mitigate it. J Infect Dis. (2019) 220:S62–S73. 10.1093/infdis/jiz214 31430386

[50] Forhan M Salas XR. Inequities in healthcare: a review of bias and discrimination in obesity treatment. Can J Diabetes. (2013) 37:205–9. 10.1016/j.jcjd.2013.03.362 24070845

[51] Guindo LA Wagner M Baltussen R Rindress D van Til J Kind P From efficacy to equity: literature review of decision criteria for resource allocation and healthcare decisionmaking. Cost Eff Resour Alloc. (2012) 10:9. 10.1186/1478-7547-10-9 22808944 PMC3495194

[52] Basu S Andrews J Kishore S Panjabi R Stuckler D. Comparative performance of private and public healthcare systems in low-and middle-income countries: a systematic review. PLoS Med. (2012) 9:e1001244. 10.1371/journal.pmed.1001244 22723748 PMC3378609

[53] Obermeyer Z Powers B Vogeli C Mullainathan S. Dissecting racial bias in an algorithm used to manage the health of populations. Science. (2019) 366:447–53. 10.1126/science.aax2342 31649194

[54] Park Y Hu J Singh M Sylla I Dankwa-Mullan I Koski E Comparison of methods to reduce bias from clinical prediction models of postpartum depression. JAMA Netw Open. (2021) 4:e213909. 10.1001/jamanetworkopen.2021.3909 33856478 PMC8050742

[55] Thompson HM Sharma B Bhalla S Boley R McCluskey C Dligach D Bias and fairness assessment of a natural language processing opioid misuse classifier: detection and mitigation of electronic health record data disadvantages across racial subgroups. J Am Med Inform Assoc. (2021) 28:2393–403. 10.1093/jamia/ocab148 34383925 PMC8510285

[56] Chen IY Pierson E Rose S Joshi S Ferryman K Ghassemi K. Ethical machine learning in healthcare. Annu Rev Biomed Data Sci. (2020) 4:123–44. 10.1146/annurev-biodatasci-092820-114757 PMC8362902 34396058

[57] Parikh RB Teeple S Navathe AS. Addressing bias in artificial intelligence in health care. JAMA. (2019) 322:2377–8. 10.1001/jama.2019.18058 31755905

[58] Fletcher RR Nakeshimana A Olubeko O. Addressing fairness, bias, and appropriate use of artificial intelligence and machine learning in global health. Front Artif Intell. (2021) 3:561802. 10.3389/frai.2020.561802 33981989 PMC8107824

[59] Pogatzki-Zahn EM Hiltrud L Lone H Winfried M Claudia W Rolf-Detlef T Developing consensus on core outcome domains for assessing effectiveness in perioperative pain management: results of the PROMPT/IMI-PainCare Delphi Meeting. Pain. (2021) 162:2717–36. 10.1097/j.pain.0000000000002254 34181367

[60] Chen Q Chen E Qian X. A narrative review on perioperative pain management strategies in enhanced recovery pathways-the past, present and future. J Clin Med. (2021) 10:2568. 10.3390/jcm10122568 34200695 PMC8229260

