# Supplementary material for: Fairness in the prediction of acute postoperative pain using machine learning models
Source: Front Digit Health. 2023 Jan 11;4:970281. doi: 10.3389/fdgth.2022.970281 (PMC9874861; doi:10.3389/fdgth.2022.970281)
Supplement: Supplementary file 1 [file Datasheet1.docx]

***Supplementary Material***

**1 Supplemental Methods. Fairness.**

**Data Cleaning Steps**

We used 113 independent variables (predictors) in the classification models, including 100 diagnosis categories. Supplemental Table S2 shows regroupings for categorical variables with groups that had small size, used for model development and validation. Supplemental Table S3 shows further regrouping for age and insurance types for fairness analyses.

Missing values for categorical variables of race, marital status, health literacy, and clinical classification software–current procedure terminology (CCS-CPT) were considered as a separate level of the variable in model training and development. Missing values for numerical variables of body mass index (BMI), area deprivation index (ADI), American Society of Anesthesiologists physical status (ASA-PS), and combined comorbidity were imputed using the average of the specific variable in the training dataset. The numerical variables were standardized, and the categorical variables were one-hot encoded (Supplemental Figure S1).

**Machine Learning Models:**

CatBoost classification model is a machine learning model that used gradient boosting on decision trees. During the training, a set of decision trees are built consecutively, and each successive tree is built with reduced loss compared to the previous trees^1,2^.

The grid search provided to the cross-validation step for CatBoost classification models included the following parameters:^1,2^

- Iterations (maximum number of trees that can be built when solving machine learning problems): 200, 400, 1000
- Depth (depth of the tree): 2, 4, 6, 8
- Learning rate (used for reducing the gradient step): 0.001, 0.01, 0.1
- Objective (the metric to use in training): Logloss

**Model Development and Evaluation:**

We used nested cross-validation with four folds for outer and inner folds. In each outer fold, 25% of the dataset was kept for testing the final model of this fold, and 75% was used for developing the model. Next, another three-fold cross-validation was performed inside each outer fold for parameter tuning, where two-thirds of the development data are used for training the model, and one-third are used for testing the model on this validation data. The missing values for the numerical variables were imputed and the numerical variables were standardized. The categorical variables were transformed into binary variables using one-hot encoding.

Optimal model candidates were selected based on the models’ area under the curve (AUC) for each combination of the parameters possible based on the model space. The final performance of the classification model (CatBoost) was tested based on the model’s predictions for the unseen test data in each fold in terms of selected model performance metrics (accuracy, AUC, precision, recall, and F1-score). The positive event was the high pain class.

**References**

1. Dorogush AV, Ershov V, Gulin A. CatBoost: gradient boosting with categorical features support. *arXiv* arXiv:181011363 (2018).
2. Prokhorenkova L, Gusev G, Vorobev A, Dorogush AV, Gulin A. CatBoost: unbiased boosting with categorical features. *arXiv* arXiv:170609516 (2017).

**2 Supplementary Tables**

**Supplemental Table 1.** Factor loadings for area deprivation index for census tracts in Florida, based on ACS5 2019.

| Factor | Loading |
| --- | --- |
| Median family income | –0.877 |
| Median mortgage | –0.699 |
| Median rent | –0.660 |
| Median house value | –0.656 |
| Percentage of families in poverty | 0.816 |
| Percentage of owner-occupied housing | –0.635 |
| Ratio of those making under 10K to those making over 50K | 0.797 |
| Percentage of people living below 150Pct federal poverty level | 0.914 |
| Percentage of households with children that are single parent | 0.706 |
| Percentage of households with no vehicle | 0.648 |
| Percentage of people with white-collar jobs | –0.822 |
| Percentage people unemployed | 0.537 |
| Percentage of people with at least HS education | –0.795 |
| Percentage of people with less than 9th-grade education | 0.646 |
| Percentage of households with over one person per room | 0.511 |

*ACS5 2019,* American Community Survey 2019 data (5-year span).

**Supplemental Table 2.** Categorical variables regrouping.

| **Variable** | **Regrouping** |
| --- | --- |
| Race:  - White  - Other (Non-white)  - American Indian  - Asian  - Black  - Hispanic  - Multiracial  - Pacific Islander  - Other  - Unknown | - ‘white’  - ‘American Indian’, ‘Asian’, ‘Black’, ‘Hispanic’, ‘Multiracial’, ‘Pacific Islander’, ‘Other’🡪 ‘non-white’  - ‘unknown’ |
| Marital status:  - Married  - Significant other  - Separated  - Widowed  - Single  - Unknown  - Other  - ? | - ‘married’, ‘life partner/significant other’🡪 ‘married’  - ‘separated’, ‘divorced’🡪 ‘divorced’  - ‘single’  - ‘widowed’  -‘?’, ‘unknown’, ‘other’🡪 ‘unknown’ |
| Health literacy:  - Adequate  - Limited  - Unable to assess | - ‘adequate’  - ‘limited’  - ‘Permanently unable to assess’, ‘Temporarily unable to assess’, ‘Unable to assess’, ‘Limited English Proficiency/Non-English Speaking Patients’, ‘Missing’🡪 ‘Unable to assess’ |
| CCS-CPT:  - Arthroplasty knee  - Arthroplasty other than hip or knee  - Debridement of wound, infection or burn  - Hip replacement, total and partial  - Incision and drainage, skin and subcutaneous tissue  - Other diagnostic procedures on musculoskeletal system  - Other fracture and dislocation procedure  - Other therapeutic procedures on muscles and tendons  - Partial excision bone  - Treatment, fracture or dislocation of hop and femur  - Treatment, fracture or dislocation of lower extremity (other than hip or femur)  - Treatment, fracture or dislocation of radius and ulna  - 'Arthroscopy',  - 'Arthrocentesis',  - 'Bone marrow biopsy',  - 'Other diagnostic radiology and related techniques',  - 'Other diagnostic procedures on musculoskeletal system'  - 'Other OR therapeutic procedures on bone',  - 'Bunionectomy or repair of toe deformities'  - 'Other OR therapeutic procedures on joints',  - 'Excision of semilunar cartilage of knee',  - 'Division of joint capsule, ligament or cartilage'  - 'Decompression peripheral nerve',  - 'Excision of skin lesion', 'Skin graft',  - 'Other OR procedures on vessels other than head and neck',  - 'Other OR therapeutic nervous system procedures',  - 'Suture of skin and subcutaneous tissue',  - 'Traction, splints, and other wound care',  -'Other therapeutic procedures on eyelids, conjunctiva, cornea',  - 'Other therapeutic procedures, hemic and lymphatic system'  - 'Spinal fusion',  - 'Laminectomy, excision intervertebral disc'  - ‘Missing’ | - Arthroplasty knee  - Arthroplasty other than hip or knee  - Debridement of wound, infection or burn  - Hip replacement, total and partial  - Incision and drainage, skin and subcutaneous tissue  - Other diagnostic procedures on musculoskeletal system  - Other fracture and dislocation procedure  - Other therapeutic procedures on muscles and tendons  - Partial excision bone  - Treatment, fracture or dislocation of hop and femur  - Treatment, fracture or dislocation of lower extremity (other than hip or femur)  - Treatment, fracture or dislocation of radius and ulna  - 'Arthroscopy', 'Arthrocentesis', 'Bone marrow biopsy',  'Other diagnostic radiology and related techniques', 'Other diagnostic procedures on musculoskeletal system'🡪"Other diagnostic procedures on musculoskeletal system"  -'Other OR therapeutic procedures on bone', 'Bunionectomy or repair of toe deformities'🡪"Other OR therapeutic procedures on bone"  -'Other OR therapeutic procedures on joints', 'Excision of semilunar cartilage of knee', 'Division of joint capsule, ligament or cartilage'🡪"Other OR therapeutic procedures on joints"  -'Decompression peripheral nerve', 'Excision of skin lesion', 'Skin graft', 'Other OR procedures on vessels other than head and neck', 'Other OR therapeutic nervous system procedures', 'Suture of skin and subcutaneous tissue', 'Traction, splints, and other wound care', 'Other therapeutic procedures on eyelids, conjunctiva, cornea', 'Other therapeutic procedures, hemic and lymphatic system'🡪"Other therapeutic procedure on skin, nerves, vessels, and lymphatics"  -'Spinal fusion', 'Laminectomy, excision intervertebral disc'🡪 "Spinal surgery (Laminectomy, fusion)"  - ‘Missing’ |

**Supplemental Table 3.** Regroupings for age and insurance for fairness analysis.

| **Attribute** | **Regrouping for fairness analysis** |
| --- | --- |
| Age | -Younger than 40 years old🡪 ‘Younger adult’  -Between 40 and 64🡪’Middle-aged’  -Older than 64 years🡪’Older adult’ |
| Insurance: | - ‘Federal’, ‘Blue Cross’, ‘Commercial’, ‘Managed Care’, ‘Medicare HMO’, ‘workers Comp’ 🡪’Private’  - ‘Medicaid’, ’Medicaid HMO’, ‘Self pay’, ‘Other’🡪’Public’  - ‘Medicare’🡪 ‘Medicare’ |

**Supplemental Table 4.** Cohort description.

| **Variable** | **All patients (N=14,263)** | **Low Pain POD1 (N=5,581)** | **High Pain POD1 (N=8,862)** | ***P v*alue** |
| --- | --- | --- | --- | --- |
| Age, mean (SD) | 60.72 (16.03) | 66.57 (14.46) | 56.96 (15.86) | <0.0001 |
| Sex:  - Female, N (%)  - Male, N (%) | 7683 (53.87%)  6580 (46.13%) | 2991 (53.59%)  2590 (46.41%) | 4692 (54.04%)  3990 (45.96%) | 0.6105 |
| Race, N (%) ^a^:  - White  - Non-white | 11405 (79.96%)  2434 (17.07%) | 4610 (82.60%)  786 (14.08%) | 6795 (78.27%)  1648 (18.98%) | <0.0001 |
| Marital status, N (%) ^b^:  - Married  - Divorced  - Widowed  - Single | 6754 (47.35%)  1405 (9.85%)  1180 (8.27%)  3772 (26.45%) | 3073 (55.06%)  427 (7.65%)  541 (9.69%)  1106 (19.82%) | 3681 (42.40%)  978 (11.26%)  639 (7.36%)  2666 (30.71%) | <0.0001 |
| Language, N (%):  - English speaking  - Non-English speaking | 14034 (98.39 %)  229 (1.61%) | 5472 (98.05%)  109 (1.95%) | 8562 (98.62%)  120 (1.38%) | 0.0099 |
| Ethnicity, N (%) ^c^:  - Hispanic  - Not_hispanic | 456 (3.20%)  13315 (93.35%) | 159 (2.85%)  5214 (93.42%) | 297 (3.42%)  8101 (93.31%) | 0.0722 |
| BMI^d^, mean (sd) | 30.08 (7.40) | 29.73 (6.92) | 30.30 (7.68) | <0.0001 |
| ADI^e^, mean (sd) | 103.35 (15.40) | 100.92 (15.42) | 104.92 (15.17) | <0.0001 |
| Insurance, N (%):  - Blue cross  - Commercial  - Federal  - Managed care  - Medicaid  - Medicaid HMO  - Medicare  - Medicare HMO  - Self pay  - Workers comp  - Other | 2080 (14.58%)  550 (3.86%)  250 (1.75%)  1006 (7.05%)  480 (3.37%)  1133 (7.94%)  5583 (39.14%)  1995 (13.99%)  831 (5.83%)  226 (1.58%)  129 (0.90%) | 831 (14.89%)  132 (2.37%)  105 (1.88%)  401 (7.19%)  99 (1.77%)  183 (3.28%)  2724 (48.81%)  869 (15.57%)  150 (2.69%)  56 (1.00%)  31 (0.56%) | 1249 (14.39%)  418 (4.81%)  145 (1.67%)  605 (6.97%)  381 (4.39%)  950 (10.94%)  2859 (32.93%)  1126 (12.97%)  681 (7.84%)  170 (1.96%)  98 (1.13%) | <0.0001 |
| Health literacy, N (%) ^f^:  - Adequate  - Limited | 10223 (71.67%)  1317 (9.23%) | 3957 (70.90%)  494 (8.85%) | 6266 (72.17%)  823 (9.48%) | 0.4179 |
| Disease categories, N (%):  - Bacterial infection; unspecified site  - Hepatitis  - Cancer of bone and connective tissue  - Other non-epithelial cancer of skin  - Cancer of breast  - Cancer of prostate  - Secondary malignancies  - Other and unspecified benign neoplasm  - Thyroid disorders  - Diabetes mellitus without complication  - Diabetes mellitus with complications  - Nutritional deficiencies  - Disorders of lipid metabolism  - Gout and other crystal arthropathies  - Fluid and electrolyte disorders  - Other nutritional; endocrine; and metabolic disorders  - Deficiency and other anemia  - Acute posthemorrhagic anemia  - Coagulation and hemorrhagic disorders  - Other hereditary and degenerative nervous system conditions  - Epilepsy; convulsions  - Headache; including migraine  **- Coma; stupor; and brain damage**  - Cataract  - Glaucoma  - Other eye disorders  - Other ear and sense organ disorders  - Other nervous system disorders  - Heart valve disorders  - Essential hypertension  - Hypertension with complications and secondary hypertension  - Coronary atherosclerosis and other heart disease  - Pulmonary heart disease  - Conduction disorders  - Cardiac dysrhythmias  - Congestive heart failure; nonhypertensive  - Peripheral and visceral atherosclerosis  - Other circulatory disease  - Phlebitis; thrombophlebitis and thromboembolism  - Chronic obstructive pulmonary disease and bronchiectasis  - Asthma  - Pleurisy; pneumothorax; pulmonary collapse  - Respiratory failure; insufficiency; arrest (adult)  - Other lower respiratory disease  - Other upper respiratory disease  - Esophageal disorders  - Other liver diseases  - **Other gastrointestinal disorders**  - Acute and unspecified renal failure  - Chronic kidney disease  - Urinary tract infections  - Calculus of urinary tract  - Genitourinary symptoms and ill-defined conditions  - Hyperplasia of prostate  - Skin and subcutaneous tissue infections  - Chronic ulcer of skin  - Other skin disorders  - Infective arthritis and osteomyelitis (except that caused by tuberculosis or sexually transmitted disease)  - Rheumatoid arthritis and related disease  - Osteoarthritis  - Other non-traumatic joint disorders  - Spondylosis; intervertebral disc disorders; other back problems  - Osteoporosis  - Pathological fracture  - Acquired foot deformities  - Other acquired deformities  - Other connective tissue disease  - Other bone disease and musculoskeletal deformities  - Other congenital anomalies  - Joint disorders and dislocations; trauma-related  - Fracture of neck of femur (hip)  - Fracture of upper limb  - Fracture of lower limb  - Other fractures  - Sprains and strains  - Crushing injury or internal injury  - Open wounds of head; neck; and trunk  - Open wounds of extremities  - Complication of device; implant or graft  - Complications of surgical procedures or medical care  - Superficial injury; contusion  - Other injuries and conditions due to external causes  - Allergic reactions  - Other aftercare  - Residual codes; unclassified  - **Anxiety disorders**  - Delirium, dementia, and amnestic and other cognitive disorders  - **Mood disorders**  - Alcohol-related disorders  - Substance-related disorders  - Screening and history of mental health and substance abuse codes  - E Codes: Cut/pierced  - E Codes: Fall  - E Codes: Motor vehicle traffic (MVT)  - E Codes: Transport; not MVT  - E Codes: Natural/environment  - E Codes: Struck by; against  - E Codes: Adverse effects of medical care  - E Codes: Adverse effects of medical drugs  - E Codes: Unspecified | 298 (2.09%)  197 (1.38%)  195 (1.37%)  427 (2.99%)  346 (2.43%)  173 (1.21%)  190 (1.33%)  440 (3.08%)  1555 (10.90%)  1830 (12.83%)  572 (4.01%)  398 (2.79%)  3740 (26.22%)  375 (2.63%)  758 (5.31%)  2976 (20.87%)  935 (6.56%)  913 (6.40%)  223 (1.56%)  218 (1.53%)  297 (2.08%)  487 (3.41%)  182 (1.28%)  162 (1.14%)  278 (1.95%)  235 (1.65%)  324 (2.27%)  3193 (22.39%)  393 (2.76%)  5693 (39.91%)  845 (5.92%)  1331 (9.33%)  274 (1.92%)  382 (2.68%)  1056 (7.40%)  528 (3.70%)  199 (1.40%)  979 (6.86%)  444 (3.11%)  1042 (7.31%)  1318 (9.24%)  184 (1.29%)  289 (2.03%)  356 (2.50%)  470 (3.30%)  3680 (25.80%)  232 (1.63%)  816 (5.72%)  320 (2.24%)  815 (5.71%)  255 (1.79%)  144 (1.01%)  509 (3.57%)  255 (1.79%)  376 (2.64%)  236 (1.65%)  386 (2.71%)  379 (2.66%)  346 (2.43%)  4232 (29.67%)  1782 (12.49%)  1145 (8.03%)  955 (6.70%)  178 (1.25%)  295 (2.07%)  905 (6.35%)  4576 (32.08%)  1330 (9.32%)  181 (1.27%)  1105 (7.75%)  505 (3.54%)  1347 (9.44%)  1124 (7.88%)  421 (2.95%)  1037 (7.27%)  207 (1.45%)  175 (1.23%)  464 (3.25%)  1296 (9.09%)  621 (4.35%)  341 (2.39%)  466 (3.27%)  3351 (23.49%)  4565 (32.01%)  3890 (27.27%)  2131 (14.94%)  317 (2.22%)  2275 (15.95%)  294 (2.06%)  1499 (10.51%)  3994 (28.00%)  149 (1.04%)  1775 (12.44%)  614 (4.30%)  149 (1.044%)  737 (5.17%)  196 (1.37%)  1486 (10.42%)  203 (1.42%)  1100 (7.71%) | 123 (2.20%)  77 (1.38%)  83 (1.49%)  185 (3.31%)  139 (2.49%)  79 (1.42%)  67 (1.20%)  173 (3.10%)  635 (11.38%)  705 (12.63%)  204 (3.66%)  147 (2.63%)  1452 (26.02%)  142 (2.54%)  322 (5.77%)  1157 (20.73%)  346 (6.20%)  368 (6.59%)  76 (1.36%)  96 (1.72%)  121 (2.17%)  195 (3.49%)  85 (1.52%)  59 (1.06%)  114 (2.04%)  92 (1.65%)  129 (2.31%)  1266 (22.68%)  162 (2.90%)  2244 (40.21%)  338 (6.06%)  524 (9.39%)  103 (1.85%)  140 (2.51%)  429 (7.69%)  197 (3.53%)  84 (1.51%)  384 (6.88%)  173 (3.10%)  422 (7.56%)  528 (9.46%)  80 (1.43%)  115 (2.06%)  126 (2.26%)  180 (3.23%)  1458 (26.12%)  80 (1.43%)  291 (5.21%)  127 (2.28%)  336 (6.02%)  103 (1.85%)  56 (1.00%)  207 (3.71%)  105 (1.88%)  145 (2.60%)  93 (1.67%)  144 (2.58%)  134 (2.40%)  125 (2.24%)  1659 (29.73%)  674 (12.08%)  459 (8.22%)  371 (6.65%)  62 (1.11%)  111 (1.99%)  361 (6.47%)  1785 (31.98%)  529 (9.48%)  68 (1.22%)  441 (7.90%)  209 (3.74%)  498 (8.92%)  442 (7.92%)  159 (2.85%)  397 (7.11%)  73 (1.31%)  70 (1.25%)  177 (3.17%)  503 (9.01%)  233 (4.17%)  129 (2.31%)  203 (3.64%)  1307 (23.42%)  1749 (31.34%)  1494 (26.77%)  788 (14.12%)  139 (2.49%)  826 (14.80%)  124 (2.22%)  565 (10.12%)  1512 (27.09%)  67 (1.20%)  706 (12.65%)  228 (4.09%)  65 (1.16%)  284 (5.09%)  67 (1.20%)  582 (10.43%)  66 (1.18%)  426 (7.63%) | 175 (2.02%)  120 (1.38%)  112 (1.29%)  241 (2.78%)  207 (2.38%)  94 (1.08%)  123 (1.42%)  267 (3.08%)  920 (10.60%)  1125 (12.96%)  368 (4.24%)  251 (2.89%)  2288 (26.35%)  233 (2.68%)  436 (5.02%)  1819 (20.95%)  589 (6.78%)  545 (6.28%)  147 (1.69%)  122 (1.41%)  176 (2.03%)  292 (3.36%)  97 (1.12%)  103 (1.19%)  164 (1.89%)  143 (1.65%)  195 (2.25%)  1927 (22.20%)  231 (2.66%)  3449 (39.73%)  507 (5.84%)  807 (9.30%)  171 (1.97%)  242 (2.79%)  627 (7.22%)  331 (3.81%)  115 (1.32%)  595 (6.85%)  271 (3.12%)  620 (7.14%)  790 (9.10%)  104 (1.20%)  174 (2.00%)  230 (2.65%)  290 (3.34%)  2222 (25.59%)  152 (1.75%)  525 (6.05%)  193 (2.22%)  479 (5.52%)  152 (1.75%)  88 (1.01%)  302 (3.48%)  150 (1.73%)  231 (2.66%)  143 (1.65%)  242 (2.79%)  245 (2.82%)  221 (2.55%)  2573 (29.64%)  1108 (12.76%)  686 (7.90%)  584 (6.73%)  116 (1.34%)  184 (2.12%)  544 (6.27%)  2791 (32.15%)  801 (9.23%)  113 (1.30%)  664 (7.65%)  296 (3.41%)  849 (9.78%)  682 (7.86%)  262 (3.02%)  640 (7.37%)  134 (1.54%) 105 (1.21%)  287 (3.31%)  793 (9.13%)  388 (4.47%)  212 (2.44%)  263 (3.03%)  2044 (23.54%)  2816 (32.43%)  2396 (27.60%)  1343 (15.47%)  178 (2.05%)  1449 (16.69%)  170 (1.96%)  934 (10.76%)  2482 (28.59%)  82 (0.94%)  1069 (12.31%)  386 (4.45%)  84 (0.97%)  453 (5.22%)  129 (1.49%)  904 (10.41%)  137 (1.58%)  674 (7.76%) | 0.4795  1.00  0.3598  0.0726  0.7285  0.0903  0.3056  0.9738  0.1517  0.5878  0.0912  0.3910  0.6698  0.6498  0.0568  0.7680  0.1796  0.4725  0.1368  0.1538  0.6065  0.7097  **0.0423**  0.5289  0.5580  1.00  0.8429  0.5074  0.4183  0.5781  0.6182  0.8739  0.6425  0.3403  0.3162  0.4082  0.4100  0.9770  0.9816  0.3638  0.4854  0.2540  0.8630  0.1592  0.7433  0.4915  0.1632  **0.0400**  0.8815  0.2199  0.7247  1.00  0.4977  0.5411  0.8618  0.9834  0.4893  0.1410  0.2702  0.9237  0.2371  0.5085  0.8808  0.2692  0.6356  0.6534  0.8527  0.6335  0.7217  0.6022  0.3117  0.0937  0.9144  0.5957  0.5848  0.2821  0.8732  0.6946  0.8292  0.4248  0.6589  0.0517  0.8803  0.1765  0.2872  **0.0291**  0.0924  **0.0028**  0.3070  0.2390  0.0545  0.1666  0.5690  0.3205  0.2957  0.7635  0.1755  0.9982  .0610  0.8009 |
| CCS-CPT category, N (%) ^g^:  - Amputation of lower extremity  - Arthroplasty knee  - Arthroplasty other than hip or knee  - Debridement of wound, infection or burn  - Hip replacement, total and partial  - Incision and drainage, skin and subcutaneous tissue  - Other OR therapeutic procedures on bone  - Other therapeutic procedures on joints  - Other OR therapeutic procedures on musculoskeletal system  - Other diagnostic procedures on musculoskeletal system  - Other fracture and dislocation procedure  - Other therapeutic procedure on skin, nerves, vessels, and lymphatics  - Other therapeutic procedures on muscles and tendons  - Partial excision bone  -Spinal surgery (Laminectomy, fusion)  - Treatment, fracture or dislocation of hop and femur  - Treatment, fracture or dislocation of lower extremity (other than hip or femur)  - Treatment, fracture or dislocation of radius and ulna | 140 (0.98%)  2693 (18.88%)  1006 (7.05%)  373 (2.62%)  2666 (18.69%)  188 (1.32%)  286 (2.01%)  420 (2.94%)  56 (0.39%)  225 (1.58%)  349 (2.45%)  49 (0.34%)  455 (3.19%)  149 (1.04%)  253 (1.77%)  999 (7.00%)  914 (6.41%)  155 (1.09%) | 47 (0.84%)  1102 (19.75%)  522 (9.35%)  99 (1.77%)  1125 (20.16%)  46 (0.82%)  61 (1.09%)  129 (2.31%)  15 (0.27%)  88 (1.58%)  99 (1.77%)  18 (0.32%)  190 (3.40%)  58 (1.04%)  68 (1.22%)  429 (7.69%)  246 (4.41%)  22 (0.39%) | 93 (1.07%)  1591 (18.33%)  484 (5.57%)  274 (3.16%)  1541 (17.75%)  142 (1.64%)  225 (2.59%)  291 (3.35%)  41 (0.47%)  137 (1.58%)  250 (2.88%)  31 (0.36%)  265 (3.05%)  91 (1.05%)  185 (2.13%)  570 (6.57%)  668 (7.69%)  133 (1.53%) | <0.0001 |
| ASA-PS^h^, mean (sd) | 2.75 (0.58) | 2.77 (0.56) | 2.74 (0.60) | 0.0005 |
| Combined comorbidity score, mean (sd) | 0.50 (1.64) | 0.51 (1.64) | 0.50 (1.64) | 0.8139 |

^a^ Number of missing values: 424

^b^ Number of missing values: 1152

^c^ Number of missing values: 492

^d^ Number of missing values: 1247

^e^ Number of missing values: 2

^f^ Number of missing values and unable to assess: 2723

^g^ Number of missing values: 2887

^h^ Number of missing values: 2473

*ASA-PS* American Society of Anesthesiologists - physical status, *POD1* postoperative day 1.

**Supplemental Table 5.** Model performance for each unprivileged group compared to the privileged group.

| **Attribute** | **Group** | **Equal opportunity ratio** | **Accuracy equality ratio** | **Predictive parity ratio** | **Predictive equality ratio** | **Statistical parity ratio** |
| --- | --- | --- | --- | --- | --- | --- |
| Race & Age | White & Younger adult* (9.14%) | 1.00 | 0.82 | 0.82 | 1.00 | 1.00 |
|  | White & Middle-aged (31.29%) | 0.98 | 0.87 | 0.88 | 0.90 | 0.96 |
|  | White & Older adult (39.53%) | **0.51** | **0.75** | **0.73** | **0.29** | **0.39** |
|  | Other & Younger adult (3.67%) | 1.00 | 0.96 | 0.96 | 1.00 | 1.00 |
|  | Other & Middle-aged (7.95%) | 0.99 | 0.90 | 0.90 | 0.97 | 0.99 |
|  | Other & Older adult (5.44%) | **0.61** | **0.76** | **0.78** | **0.36** | **0.49** |
| Sex & Age | Male & Younger adult* (7.82%) | 1.00 | 0.81 | 0.81 | 1.00 | 1.00 |
|  | Male & Middle-aged (19.86%) | 0.97 | 0.87 | 0.88 | 0.91 | 0.95 |
|  | Male & Older adult (18.45%) | **0.42** | **0.77** | **0.70** | **0.23** | **0.31** |
|  | Female & Younger adult (5.34%) | 1.00 | 1.00 | 1.00 | 1.00 | 1.00 |
|  | Female & Middle-aged (20.61%) | 0.99 | 0.89 | 0.90 | 0.92 | 0.97 |
|  | Female & Older adult (27.91%) | **0.58** | **0.76** | **0.76** | **0.34** | **0.46** |
| Language & Age | English & Younger adult* (12.84%) | 1.00 | 0.82 | 0.82 | 1.00 | 1.00 |
|  | English & Middle-aged (39.91%) | 0.98 | 0.88 | 0.89 | 0.92 | 0.96 |
|  | English & Older adult (45.65%), | **0.52** | **0.76** | **0.74** | **0.30** | **0.40** |
|  | Non-English & Younger adult (0.32%) | 1.00 | 0.88 | 0.88 | 1.00 | 1.00 |
|  | Non-English & Middle-aged (0.57%) | 0.98 | **0.76** | **0.73** | 0.83 | 0.91 |
|  | Non-English & Older adult (0.72%) | **0.31** | 0.82 | 0.88 | **0.08** | **0.18** |
| Health Literacy & Age | Adequate HL & Younger adult* (8.60%) | 1.00 | 0.82 | 0.82 | 1.00 | 1.00 |
|  | Adequate HL & Middle-aged (29.52%) | 0.98 | 0.88 | 0.89 | 0.91 | 0.96 |
|  | Adequate HL & Older adult (33.55%) | **0.56** | **0.75** | **0.73** | **0.33** | **0.44** |
|  | Limited HL & Younger adult (0.93%) | 1.00 | 0.95 | 0.95 | 1.00 | 1.00 |
|  | Limited HL & Middle-aged (3.62%) | 0.99 | 0.94 | 0.95 | 0.98 | 0.99 |
|  | Limited HL & Older adult (4.68%) | **0.53** | **0.78** | **0.79** | **0.27** | **0.39** |
| ADI & Age | Younger adult & Lowest-tertile ADI* (3.16%) | 1.00 | 0.79 | 0.79 | 1.00 | 1.00 |
|  | Younger adult & Middle-tertile ADI (4.51%) | 1.00 | 1.04 | 1.04 | 1.00 | 1.00 |
|  | Younger adult & Highest-tertile ADI (5.49%) | 1.00 | 1.03 | 1.03 | 1.00 | 1.00 |
|  | Middle-aged & Lowest-tertile ADI (11.23%) | 0.94 | 0.83 | 0.84 | 0.83 | 0.90 |
|  | Middle-aged & Middle-tertile ADI (14.44%) | 0.99 | 0.91 | 0.91 | 0.93 | 0.97 |
|  | Middle-aged & Highest-tertile ADI (14.80%) | 1.00 | 0.96 | 0.96 | 0.99 | 1.00 |
|  | Older adult & Lowest-tertile ADI (18.70%) | **0.36** | **0.78** | **0.73** | **0.19** | **0.26** |
|  | Older adult & Middle-tertile ADI (15.52%) | **0.56** | 0.80 | **0.78** | **0.30** | **0.42** |
|  | Older adult & Highest-tertile ADI (12.14%) | **0.68** | **0.77** | **0.77** | **0.47** | **0.57** |
| Insurance & Age | Younger adult & Private insurance* (6.62%) | 1.00 | 0.78 | 0.78 | 1.00 | 1.00 |
|  | Middle-aged & Private insurance (22.25%) | 0.96 | 0.83 | 0.83 | 0.88 | 0.93 |
|  | Older adult & Private insurance (13.95%) | **0.59** | 0.80 | 0.80 | **0.34** | **0.46** |
|  | Younger adult & Public insurance (5.79%) | 1.00 | 1.09 | 1.09 | 1.00 | 1.00 |
|  | Middle-aged & Public insurance (11.87%) | 1.00 | 1.05 | 1.05 | 1.00 | 1.00 |
|  | Older adult & Public insurance (0.38%) | **0.65** | 0.83 | **0.74** | **0.36** | **0.48** |
|  | Younger adult & Medicare (0.75%) | 1.00 | 1.05 | 1.05 | 1.00 | 1.00 |
|  | Middle-aged & Medicare (6.36%) | 1.00 | 1.01 | 1.01 | 1.00 | 1.00 |
|  | Older adult & Medicare (32.03%) | **0.49** | **0.79** | **0.76** | **0.27** | **0.37** |
| Sex & Race | Male & white* (36.78%) | 0.81 | 0.69 | 0.71 | 0.48 | 0.67 |
|  | Male & Other (7.89%) | 1.15 | 1.06 | 1.04 | **1.41** | **1.25** |
|  | Female & White (43.19%) | 0.99 | 0.98 | 0.99 | 1.06 | 1.01 |
|  | Female & Other (36.77%) | 1.09 | 1.02 | 1.03 | **1.41** | 1.21 |
| Language & Race | English & White* (79.58%) | 0.80 | 0.68 | 0.71 | 0.50 | 0.68 |
|  | English & Other (15.93%) | 1.13 | 1.05 | 1.05 | **1.41** | 1.24 |
|  | Non-English & White (0.38%) | 0.86 | 0.98 | 0.91 | **0.72** | **0.76** |
|  | Non-English & Other (1.14%) | 0.95 | 0.99 | 0.96 | 0.87 | 0.90 |
| Health Literacy & Race | Adequate HL & White* (58.83%) | 0.81 | 0.68 | 0.70 | 0.53 | 0.70 |
|  | Adequate HL & Other (10.83%) | 1.12 | 1.07 | 1.07 | **1.31** | 1.21 |
|  | Limited HL & White (6.42%) | 0.95 | 1.03 | 1.06 | 0.81 | 0.91 |
|  | Limited HL & Other (2.59%) | 1.12 | 1.08 | 1.07 | 1.19 | 1.17 |
| Race & ADI | White & Lowest-tertile ADI* (27.88%) | 0.67 | 0.65 | 0.67 | 0.37 | 0.53 |
|  | White & Middle-tertile ADI (28.86%) | **1.25** | 1.06 | 1.06 | **1.42** | **1.35** |
|  | White & Highest-tertile ADI (23.20%) | **1.34** | 1.10 | 1.10 | **1.76** | **1.54** |
|  | Other race & Lowest-tertile ADI (4.01%) | 1.19 | 1.01 | 1.01 | **1.46** | **1.31** |
|  | Other race & Middle-tertile ADI (4.66%) | **1.38** | 1.15 | 1.14 | **1.69** | **1.57** |
|  | Other race & Highest-tertile ADI (8.40%) | **1.40** | 1.12 | 1.12 | **2.14** | **1.70** |
| Sex & Language | Male & English* (45.39%) | 0.83 | 0.70 | 0.72 | 0.51 | 0.70 |
|  | Male & non-English (0.74%) | 1.08 | 1.00 | 0.92 | 1.05 | 1.03 |
|  | Female & English (53.00%) | 0.99 | 0.98 | 0.98 | 1.06 | 1.01 |
|  | Female & non-English (0.86%) | **0.75** | 0.90 | 0.89 | **0.72** | **0.71** |
| Sex & Health Literacy | Male & Adequate HL* (25.91%) | 0.82 | 0.69 | 0.71 | 0.52 | 0.70 |
|  | Male & Limited HL (3.81%) | 1.02 | 1.07 | 1.08 | 0.89 | 1.00 |
|  | Female & Adequate HL (32.24%) | 1.02 | 0.99 | 0.99 | 1.10 | 1.05 |
|  | Female & Limited HL (3.67%) | 0.95 | 0.98 | 1.00 | 0.95 | 0.95 |
| Sex & ADI | Male & Lowest-tertile ADI* (15.59%) | 0.67 | 0.65 | 0.66 | 0.38 | 0.53 |
|  | Male & Middle-tertile ADI (15.71%) | **1.29** | 1.10 | 1.09 | **1.43** | **1.40** |
|  | Male & Highest-tertile ADI (14.82%) | **1.36** | 1.12 | 1.12 | **1.78** | **1.57** |
|  | Female & Lowest-tertile ADI (17.50%) | 1.04 | 1.00 | 1.01 | 1.08 | 1.06 |
|  | Female & Middle-tertile ADI (18.75%) | 1.23 | 1.05 | 1.07 | **1.43** | **1.34** |
|  | Female & Highest-tertile ADI (17.61%) | **1.34** | 1.08 | 1.10 | **1.89** | **1.58** |
| Language & Health Literacy | English & Adequate HL* (71.16%) | 0.83 | 0.69 | 0.71 | 0.55 | 0.72 |
|  | English & Limited HL (9.04%) | 0.98 | 1.03 | 1.05 | 0.87 | 0.95 |
|  | Non-English & Adequate HL (0.51%) | 0.84 | 0.92 | 0.92 | 0.83 | 0.82 |
|  | Non-English & Limited HL (0.19%) | 1.00 | 0.97 | 0.83 | 0.85 | 0.87 |
| Language & ADI | English & Lowest-tertile ADI* (32.59%) | 0.69 | 0.65 | 0.67 | 0.40 | 0.55 |
|  | English & Middle-tertile ADI (33.98%) | 1.23 | 1.07 | 1.07 | **1.37** | **1.32** |
|  | English & Highest-tertile ADI (31.81%) | **1.32** | 1.10 | 1.10 | **1.77** | **1.53** |
|  | non-English & Lowest-tertile ADI (0.50%) | 0.91 | 1.01 | 0.91 | 0.82 | 0.83 |
|  | non-English & Middle-tertile ADI (0.48%) | 1.16 | 1.02 | 0.95 | 1.23 | 1.17 |
|  | non-English & Highest-tertile ADI (0.62%) | 1.15 | 1.02 | 1.03 | **1.34** | 1.24 |
| Health Literacy & ADI | Adequate HL & Lowest-tertile ADI* (25.54%) | 0.70 | 0.65 | 0.67 | 0.41 | 0.56 |
|  | Adequate HL & Middle-tertile ADI (24.57%) | 1.23 | 1.06 | 1.06 | **1.44** | **1.34** |
|  | Adequate HL & Highest-tertile ADI (21.56%) | **1.33** | 1.11 | 1.11 | **1.84** | **1.55** |
|  | Limited HL & Lowest-tertile ADI (1.82%) | 0.89 | 0.99 | 1.06 | 0.80 | 0.87 |
|  | Limited HL & Middle-tertile ADI (3.48%) | 1.17 | 1.12 | 1.15 | 1.06 | 1.20 |
|  | Limited HL & Highest-tertile ADI (3.93%) | **1.27** | 1.09 | 1.09 | **1.50** | **1.40** |
| Insurance & Language | Private insurance & English* (42.24%) | 0.87 | 0.66 | 0.33 | 0.67 | 0.79 |
|  | Private insurance & Non-English (0.58%) | 0.81 | 1.02 | 1.03 | **0.54** | **0.69** |
|  | Medicare insurance & English (38.74%) | **0.73** | 0.98 | 1.00 | **0.50** | **0.61** |
|  | Medicare insurance & Non-English (0.41%) | **0.53** | 0.97 | 1.02 | **0.30** | **0.41** |
|  | Public insurance & English (17.42%) | 1.14 | **1.26** | 1.24 | **1.47** | **1.26** |
|  | Public insurance & Non-English (0.62%) | 1.10 | 1.00 | 0.93 | 1.05 | 1.06 |
| Insurance & ADI | Private insurance & Lowest-tertile* (15.38%) | 0.78 | 0.64 | 0.65 | 0.54 | 0.67 |
|  | Private insurance & Middle-tertile (14.68%) | 1.14 | 1.03 | 1.04 | **1.32** | 1.22 |
|  | Private insurance & Highest-tertile (12.76%) | 1.19 | 1.07 | 1.07 | **1.49** | **1.32** |
|  | Public insurance & Lowest-tertile (3.14%) | 1.25 | 1.20 | 1.21 | **1.76** | **1.44** |
|  | Public insurance & Middle-tertile (6.58%) | **1.27** | **1.29** | **1.28** | **1.77** | **1.47** |
|  | Public insurance & Highest-tertile (8.31%) | **1.27** | **1.31** | **1.30** | **1.82** | **1.48** |
|  | Medicare & Lowest-tertile (14.58%) | **0.58** | 0.98 | 0.98 | **0.41** | **0.49** |
|  | Medicare & Middle-tertile (13.19%) | 0.84 | 1.04 | 1.05 | **0.60** | **0.73** |
|  | Medicare & Highest-tertile (11.37%) | 1.00 | 1.02 | 1.05 | 0.97 | 1.00 |
| Insurance & Sex | Private insurance & Male* (20.56%) | 0.86 | 0.67 | 0.68 | 0.64 | 0.78 |
|  | Private insurance & Female (22.25%) | 1.02 | 0.99 | 0.98 | 1.06 | 1.03 |
|  | Public insurance & male (9.75%) | 1.15 | 1.23 | 1.22 | **1.49** | **1.27** |
|  | Public insurance & female (8.29%) | 1.16 | 1.24 | 1.22 | **1.49** | **1.28** |
|  | Medicare & Male (15.82%) | **0.72** | 0.98 | 0.93 | **0.50** | **0.60** |
|  | Medicare & Female (23.33%) | **0.77** | 0.96 | 0.98 | **0.62** | **0.70** |
| Insurance & Health literacy | Private insurance & Adequate* (32.17%) | 0.87 | 0.66 | 0.67 | 0.68 | 0.79 |
|  | Private insurance & Limited (2.67%) | 0.99 | 1.05 | 1.06 | 0.87 | 0.96 |
|  | Public insurance & Adequate (11.67%) | 1.15 | **1.28** | **1.26** | **1.46** | 1.25 |
|  | Public insurance & Limited (2.31%) | 1.15 | 1.25 | 1.23 | **1.39** | 1.24 |
|  | Medicare & Adequate (27.83%) | **0.76** | 0.99 | 1.00 | **0.54** | **0.65** |
|  | Medicare & Limited (4.25%) | **0.71** | 0.99 | 1.02 | **0.48** | **0.60** |
| Insurance & Race | Private insurance & White* (34.12%) | 0.86 | 0.66 | 0.67 | 0.65 | 0.78 |
|  | Private insurance & Other (7.25%) | 1.05 | 1.04 | 1.05 | 1.15 | 1.10 |
|  | Public insurance & White (12.65%) | 1.15 | **1.26** | 1.25 | **1.53** | **1.28** |
|  | Public insurance & Other (5.01%) | 1.16 | 1.22 | 1.21 | **1.40** | **1.26** |
|  | Medicare & White (33.20%) | **0.70** | 0.98 | 0.99 | **0.48** | **0.59** |
|  | Medicare & Other (4.81%) | 0.88 | 1.01 | 1.06 | **0.73** | 0.83 |

The privileged group for each attribute is marked with *. The performances with bias detected are shown in bold. Younger adult: younger than 40 years old; middle-aged: between 40 and 64 years old; older adult: older than 64 years old. The numbers in parentheses show the percentage of each subcohort in the dataset.

*ADI,* area deprivation index.

**3 Supplemental Figures**


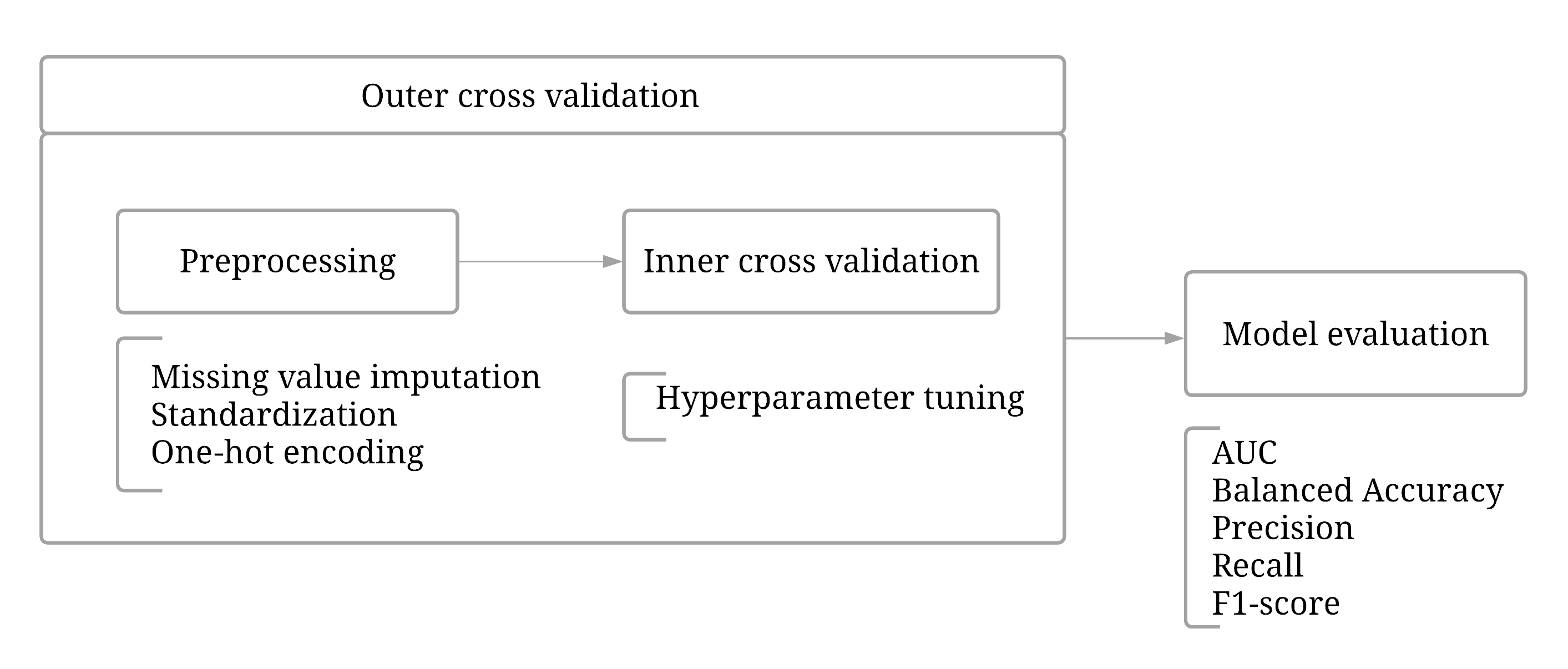


**Supplemental Figure 1.** Model development and evaluation steps.

**
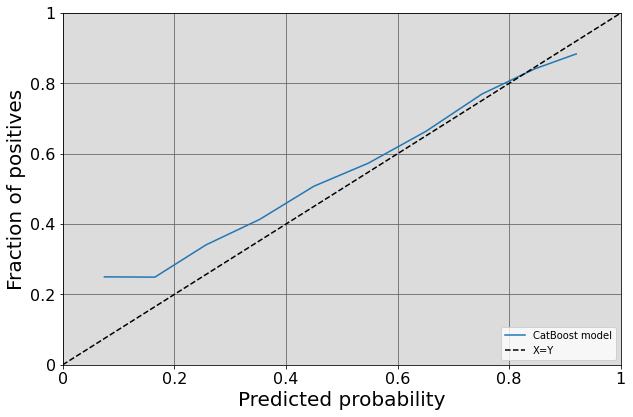
**

**Supplemental Figure 2.** Calibration plot of the CatBoost classification model used for prediction of postoperative day 1 pain level.

**
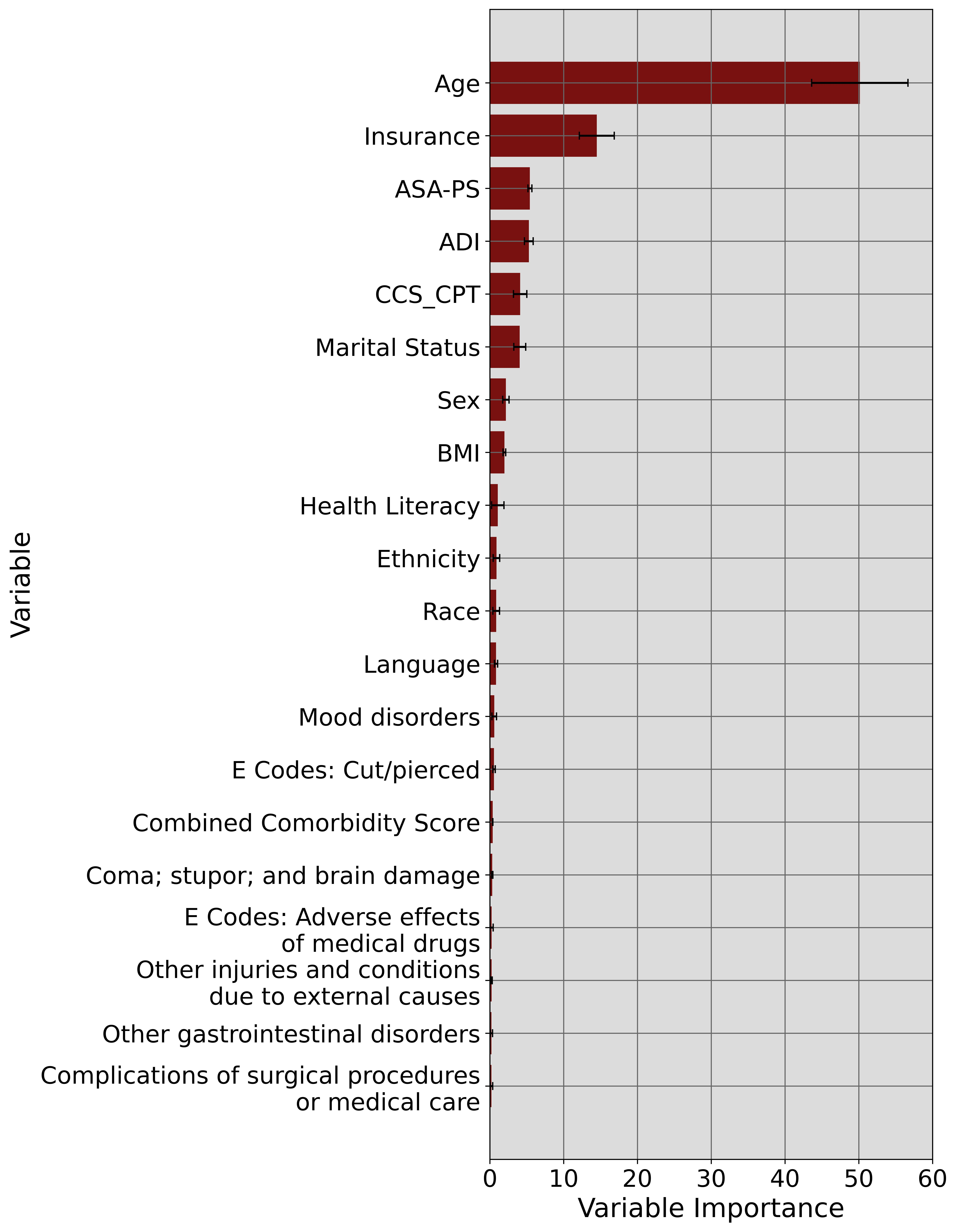
**

**Supplemental Figure 3.** Ranking of variable importance for training the CatBoost classifier using variable importance extracted from the selected model in each fold. Bar heights show the mean of the four importance values, and the error bars show the standard deviations of the four importance values for each predictor. ADI, area deprivation index; ASA-PS, American Society of Anesthesiologists physical status; BMI, body mass index; CCS-CPT, clinical classification software – current procedure terminology.


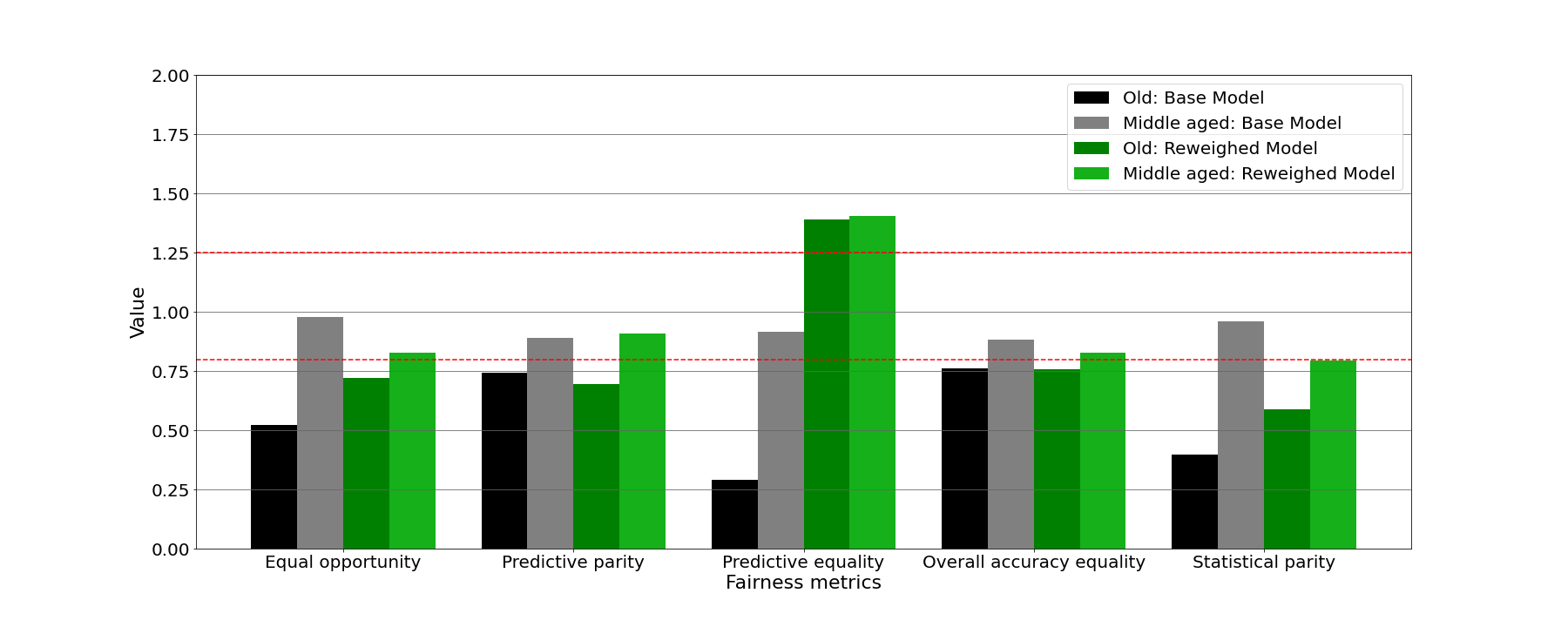


**Supplemental Figure 4.** Effect of reweighing approach on postoperative day 1 (POD1) prediction model fairness regarding age, examined as the change in fairness metrics (ratio of model performance in unprivileged groups to privileged groups). Equal opportunity: ratio of true positive rate; predictive parity: ratio of positive predictive value; predictive equality: ratio of false positive rate; statistical parity: ratio of statistical parity; overall accuracy equality: ratio of accuracy of the unprivileged subcohort to the privileged subcohort. The red horizontal lines show the predetermined thresholds for bias detection (0.8 and 1.25).


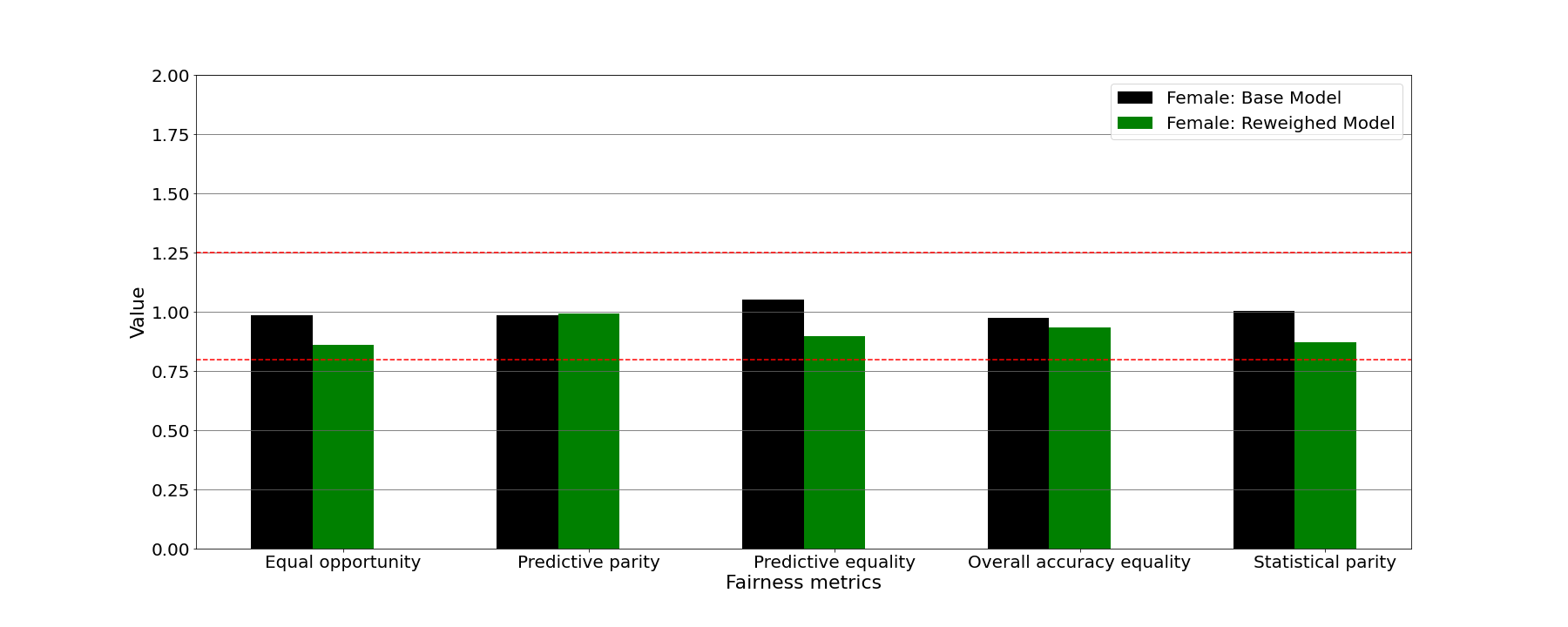


**Supplemental Figure 5.** Effect of the reweighing approach on postoperative day 1 (POD1) prediction model fairness regarding sex, examined as the change in fairness metrics (ratio of model performance in unprivileged groups to privileged groups). Equal opportunity: ratio of true positive rate; predictive parity: ratio of positive predictive value; predictive equality: ratio of false positive rate; statistical parity: ratio of statistical parity; overall accuracy equality: ratio of accuracy, of the unprivileged subcohort to the privileged subcohort. The red horizontal lines show the predetermined thresholds for bias detection (0.8 and 1.25).


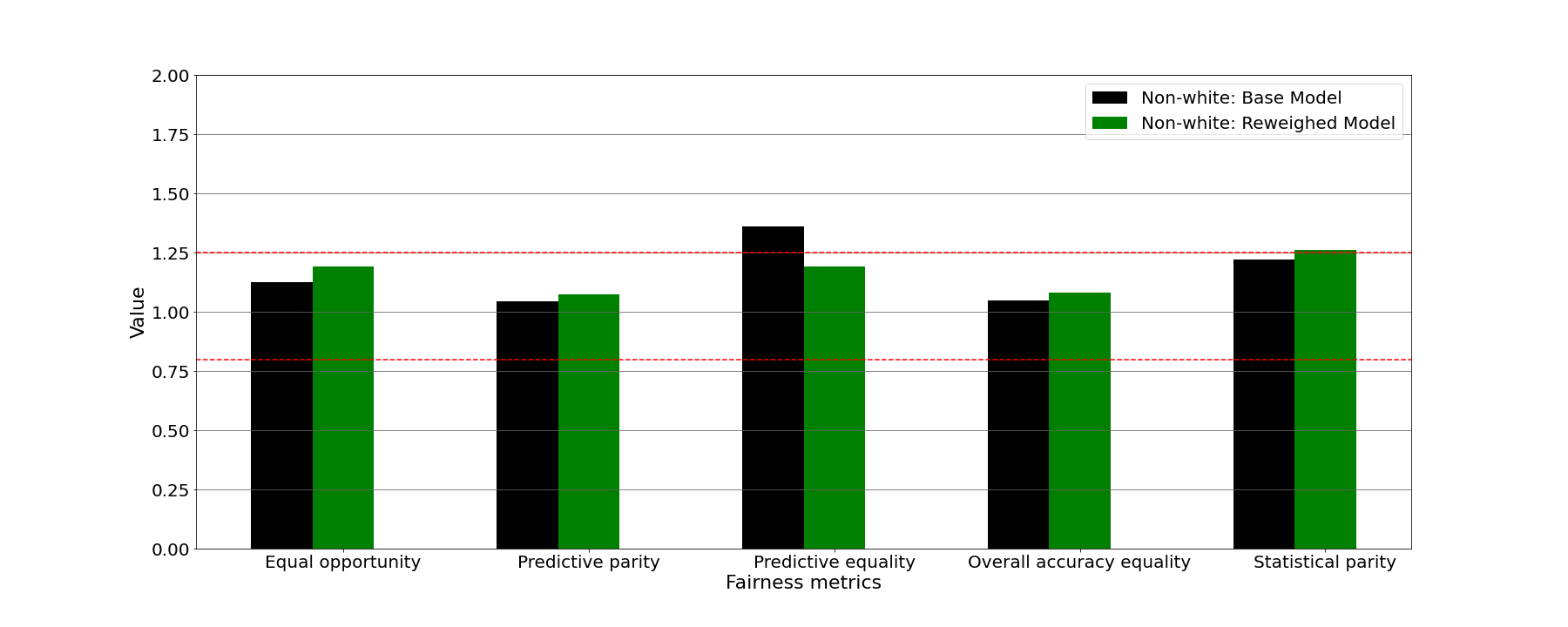


**Supplemental Figure 6.** Effect of the reweighing approach on postoperative day 1 (POD1) prediction model fairness regarding race, examined as the change in fairness metrics (ratio of model performance in unprivileged groups to privileged groups). Equal opportunity: ratio of true positive rate; predictive parity: ratio of positive predictive value; predictive equality: ratio of false positive rate; statistical parity: ratio of statistical parity; overall accuracy equality: ratio of accuracy of the unprivileged subcohort to the privileged subcohort. The red horizontal lines show the predetermined thresholds for bias detection (0.8 and 1.25).


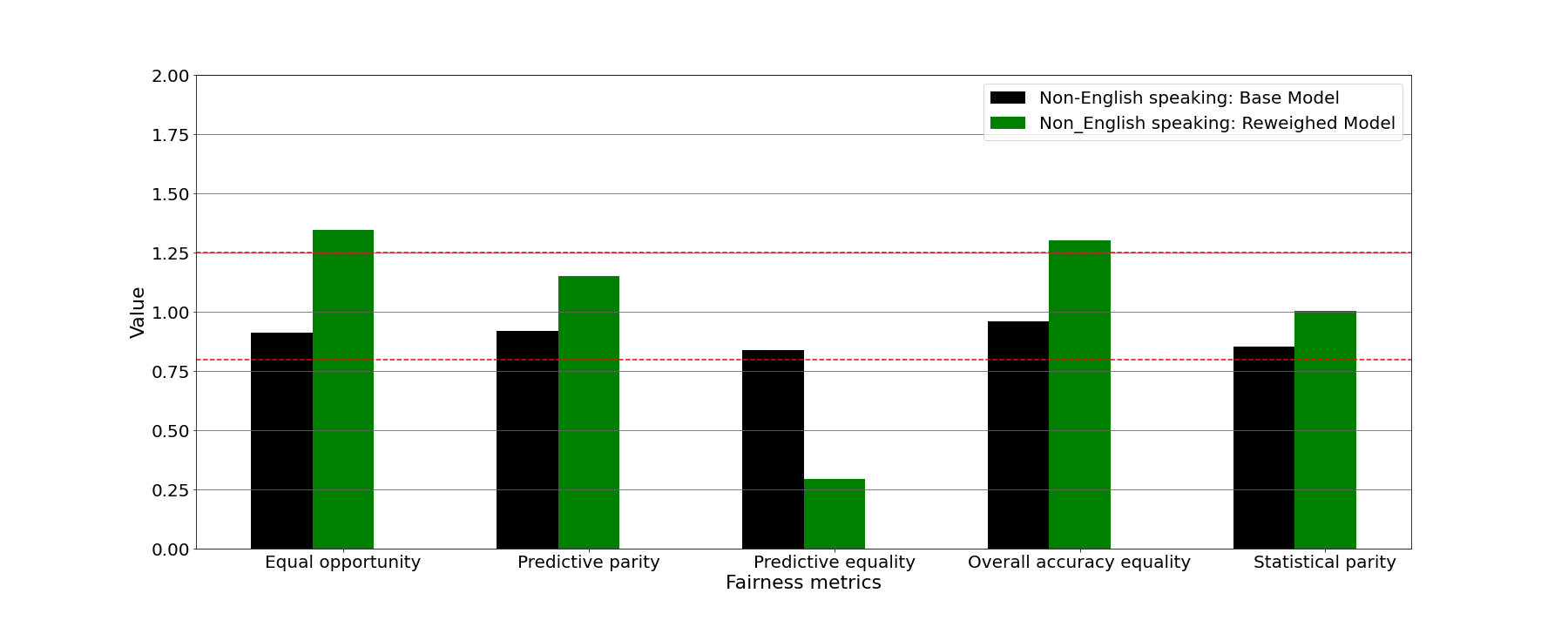


**Supplemental Figure 7.** Effect of the reweighing approach on postoperative day 1 (POD1) prediction model fairness regarding language, examined as the change in fairness metrics (ratio of model performance in unprivileged groups to privileged groups). Equal opportunity: ratio of true positive rate; predictive parity: ratio of positive predictive value; predictive equality: ratio of false positive rate; statistical parity: ratio of statistical parity; overall accuracy equality: ratio of accuracy of the unprivileged subcohort to the privileged subcohort. The red horizontal lines show the predetermined thresholds for bias detection (0.8 and 1.25).


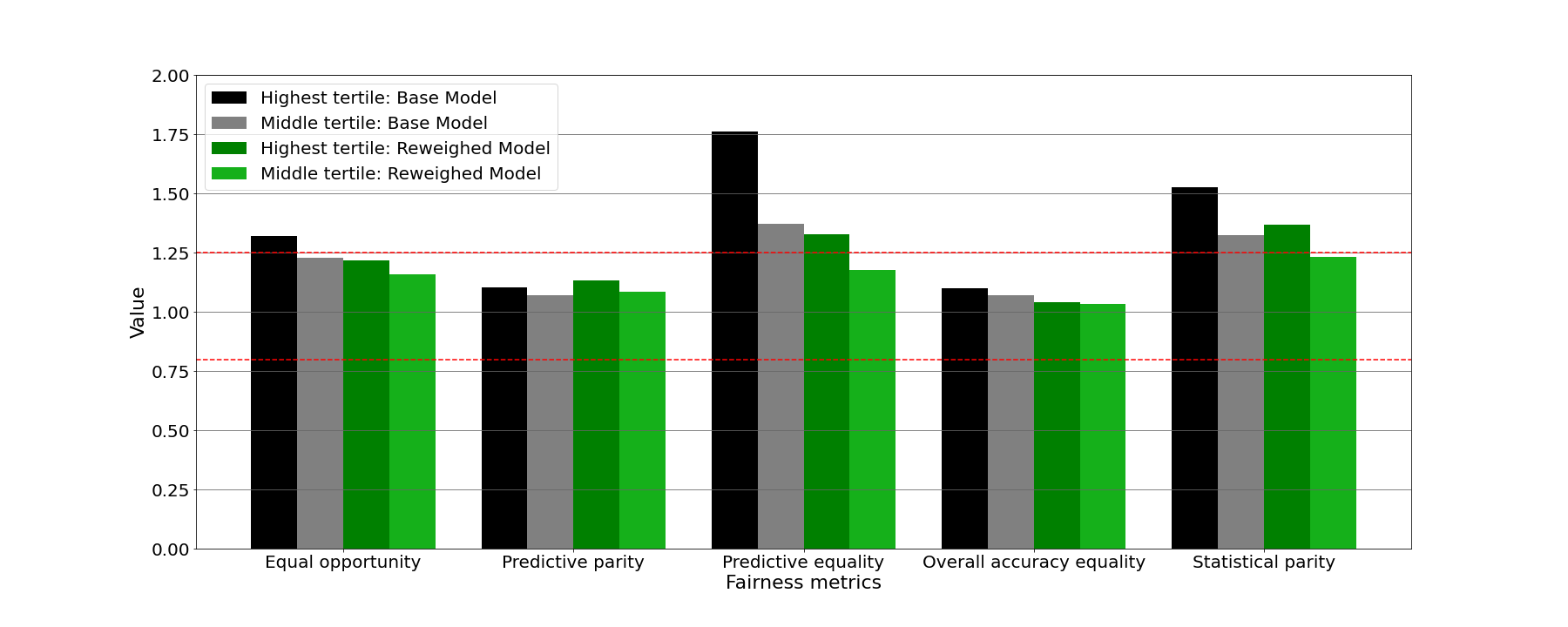


**Supplemental Figure 8.** Effect of the reweighing approach on postoperative day 1 (POD1) prediction model fairness regarding area deprivation index (ADI), examined as the change in fairness metrics (ratio of model performance in unprivileged groups to privileged groups). Equal opportunity: ratio of true positive rate; predictive parity: ratio of positive predictive value; predictive equality: ratio of false positive rate; statistical parity: ratio of statistical parity; overall accuracy equality: ratio of accuracy of the unprivileged subcohort to the privileged subcohort. The red horizontal lines show the predetermined thresholds for bias detection (0.8 and 1.25).


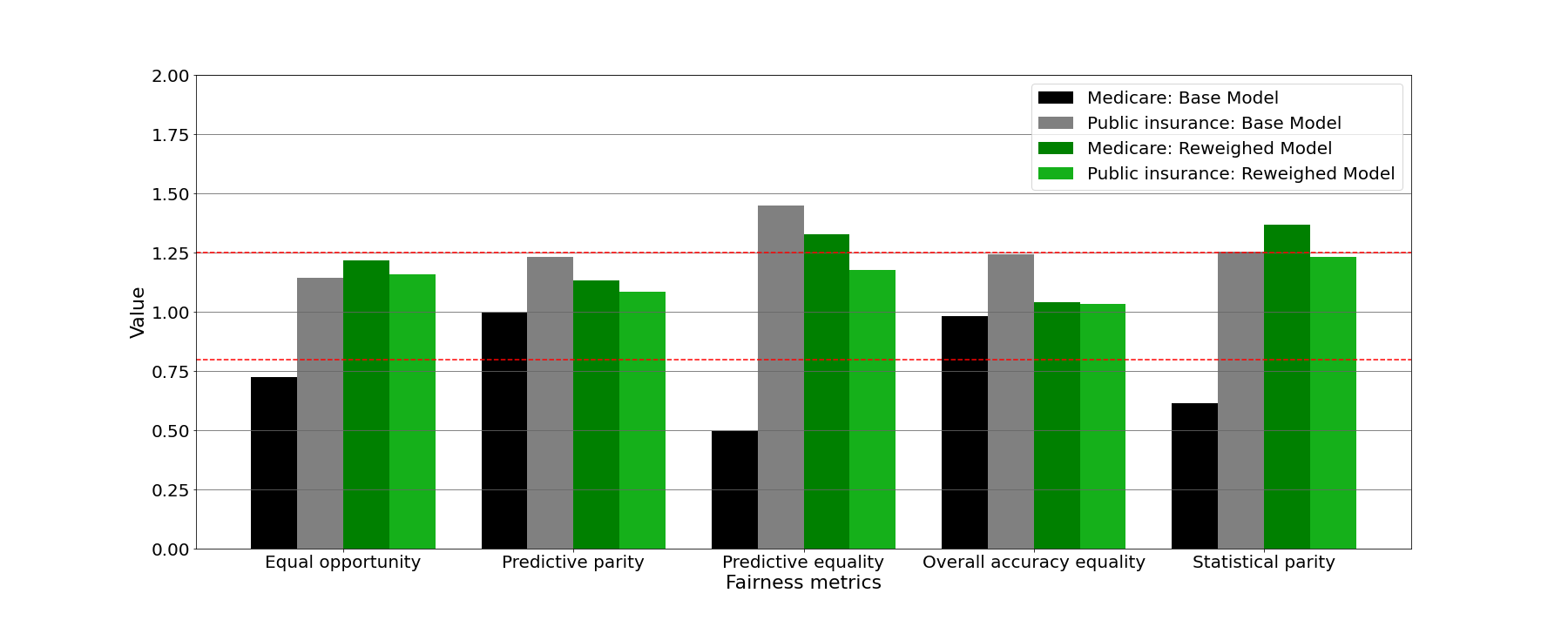


**Supplemental Figure 9.** Effect of the reweighing approach on postoperative day 1 (POD1) prediction model fairness regarding insurance type, examined as the change in fairness metrics (ratio of model performance in unprivileged groups to privileged groups). Equal opportunity: ratio of true positive rate; predictive parity: ratio of positive predictive value; predictive equality: ratio of false positive rate; statistical parity: ratio of statistical parity; overall accuracy equality: ratio of accuracy, of the unprivileged subcohort to the privileged subcohort.


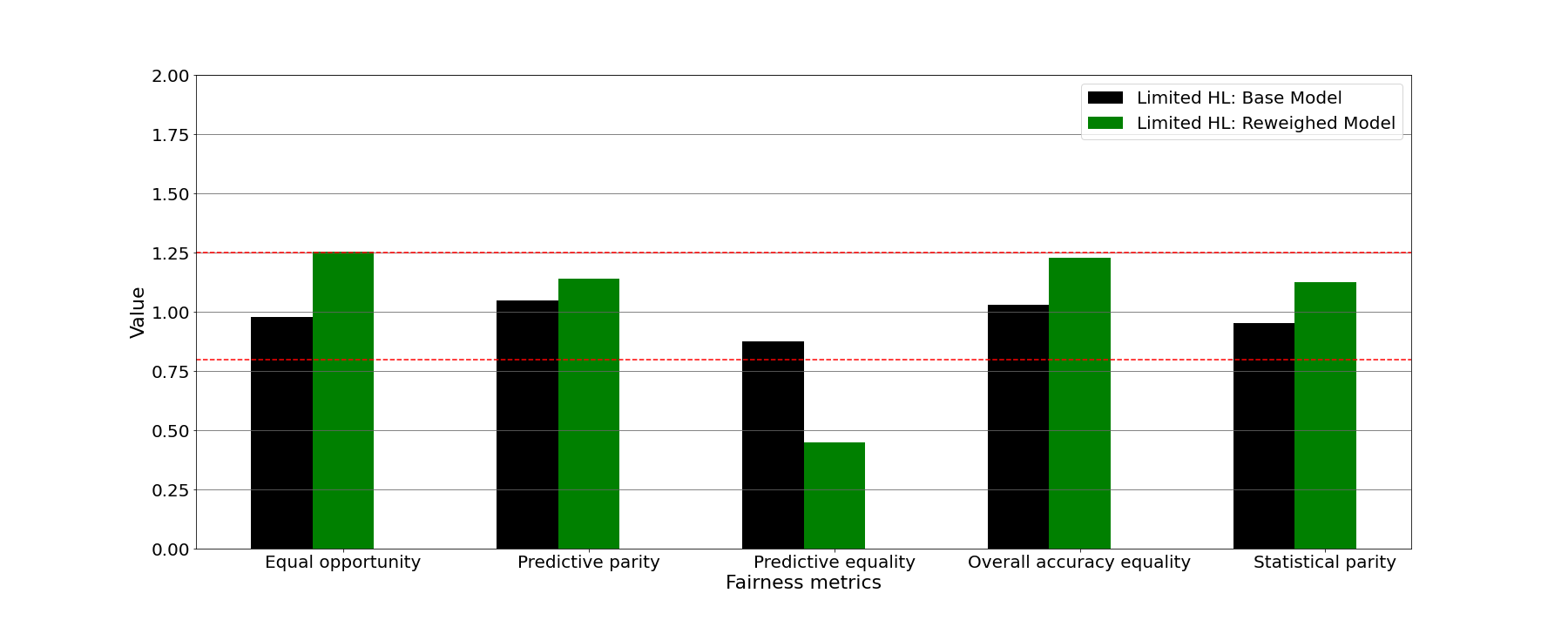


**Supplemental Figure 10.** Effect of the reweighing approach on postoperative day 1 (POD1) prediction model fairness regarding health literacy, examined as the change in fairness metrics (ratio of model performance in unprivileged groups to privileged groups). Equal opportunity: ratio of true positive rate; predictive parity: ratio of positive predictive value; predictive equality: ratio of false positive rate; statistical parity: ratio of statistical parity; overall accuracy equality: ratio of accuracy of the unprivileged subcohort to the privileged subcohort. The red horizontal lines show the predetermined thresholds for bias detection (0.8 and 1.25). *HL* health literacy.

*
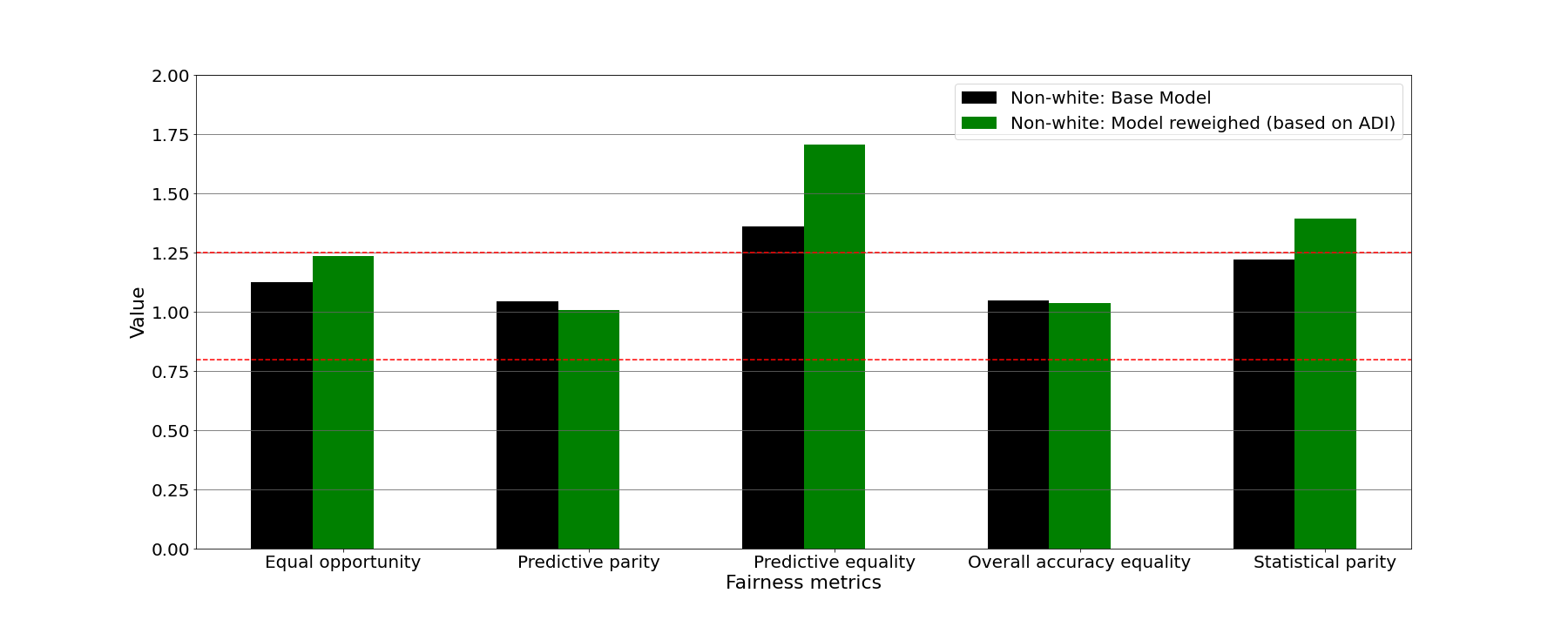
*

**Supplemental Figure 11.** Effect of reweighing approach using area deprivation index (ADI) tertile attribute on postoperative day 1 (POD1) prediction model fairness regarding race, examined as the change in fairness metrics (ratio of model performance in unprivileged groups to privileged groups). Equal opportunity: ratio of true positive rate; predictive parity: ratio of positive predictive value; predictive equality: ratio of false positive rate; statistical parity: ratio of statistical parity; overall accuracy equality: ratio of accuracy, of the unprivileged subcohort to the privileged subcohort. The red horizontal lines show the predetermined thresholds for bias detection (0.8 and 1.25).
